# Supplementary figures and images for: ciRS-7 expression is epigenetically regulated in cancer cells across human adenocarcinomas
Source: PLoS Genet. 2025 Jun 2;21(6):e1011726. doi: 10.1371/journal.pgen.1011726 (PMC12162099; doi:10.1371/journal.pgen.1011726)

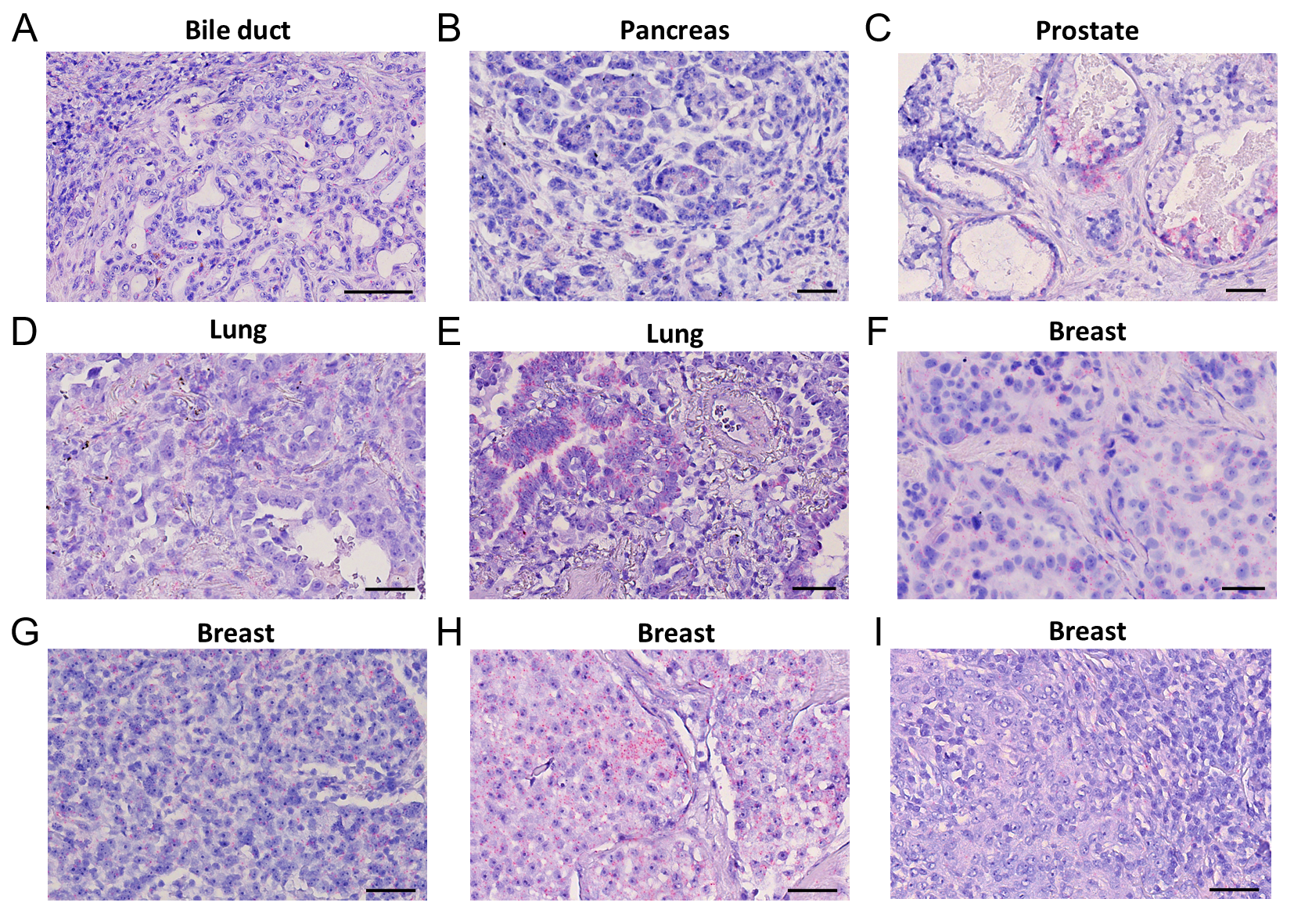

Supplement: S1 Fig — A-I) Chromogenic in situ hybridization of ciRS-7 in bile duct (A), pancreatic (B), prostate (C), lung (D, E), and breast (F-I) adenocarcinomas. ciRS-7 signals are observed as pink dots. Scale bars are indicated in the lower-left corners (A = 100 µm, B-I = 50 µm). (TIF) [file pgen.1011726.s001.tif]

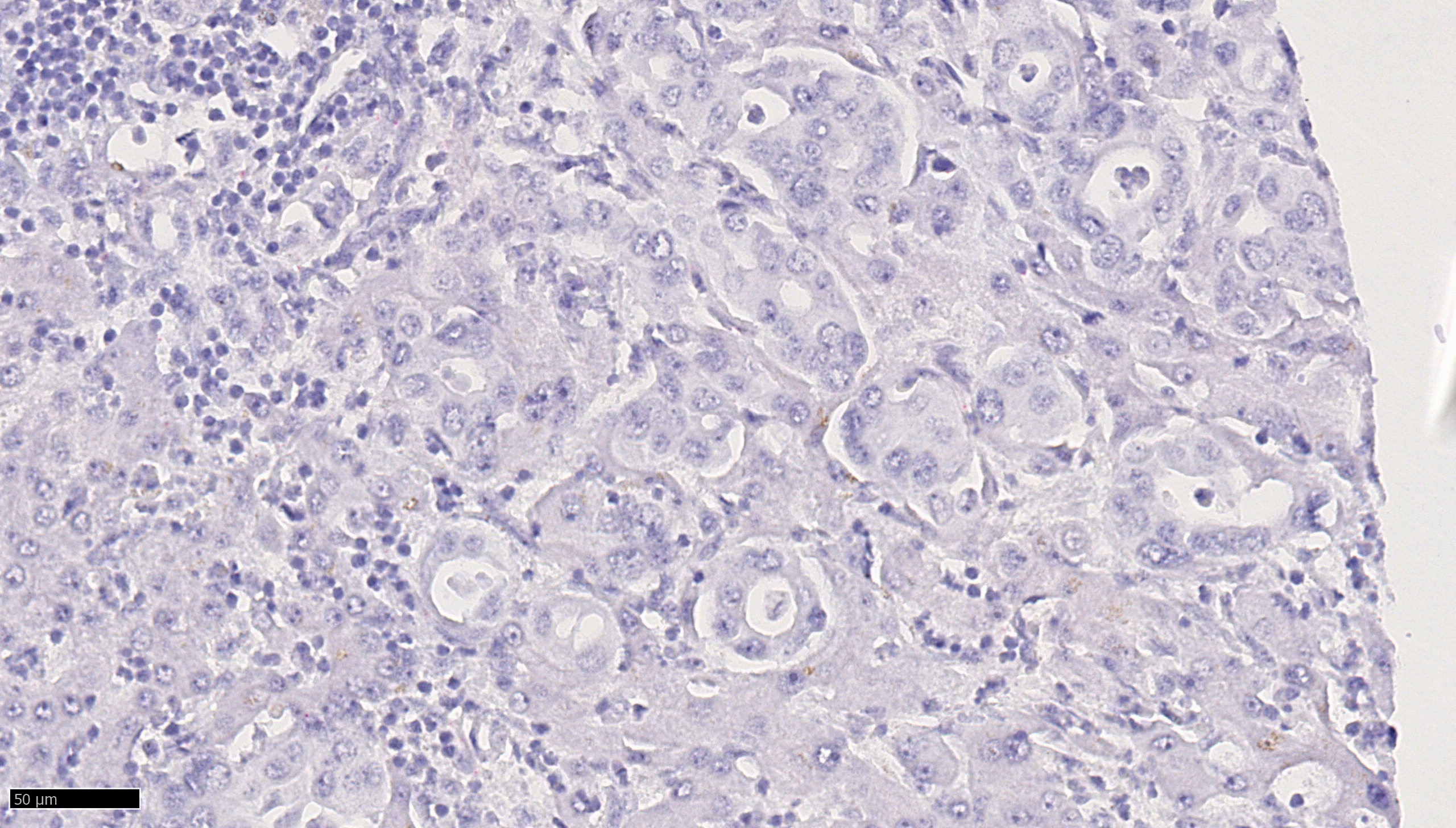

Supplement: S1 File — (ZIP) [file pgen.1011726.s002.zip › S2 figures - Kopi/Bile_duct_1.tif]

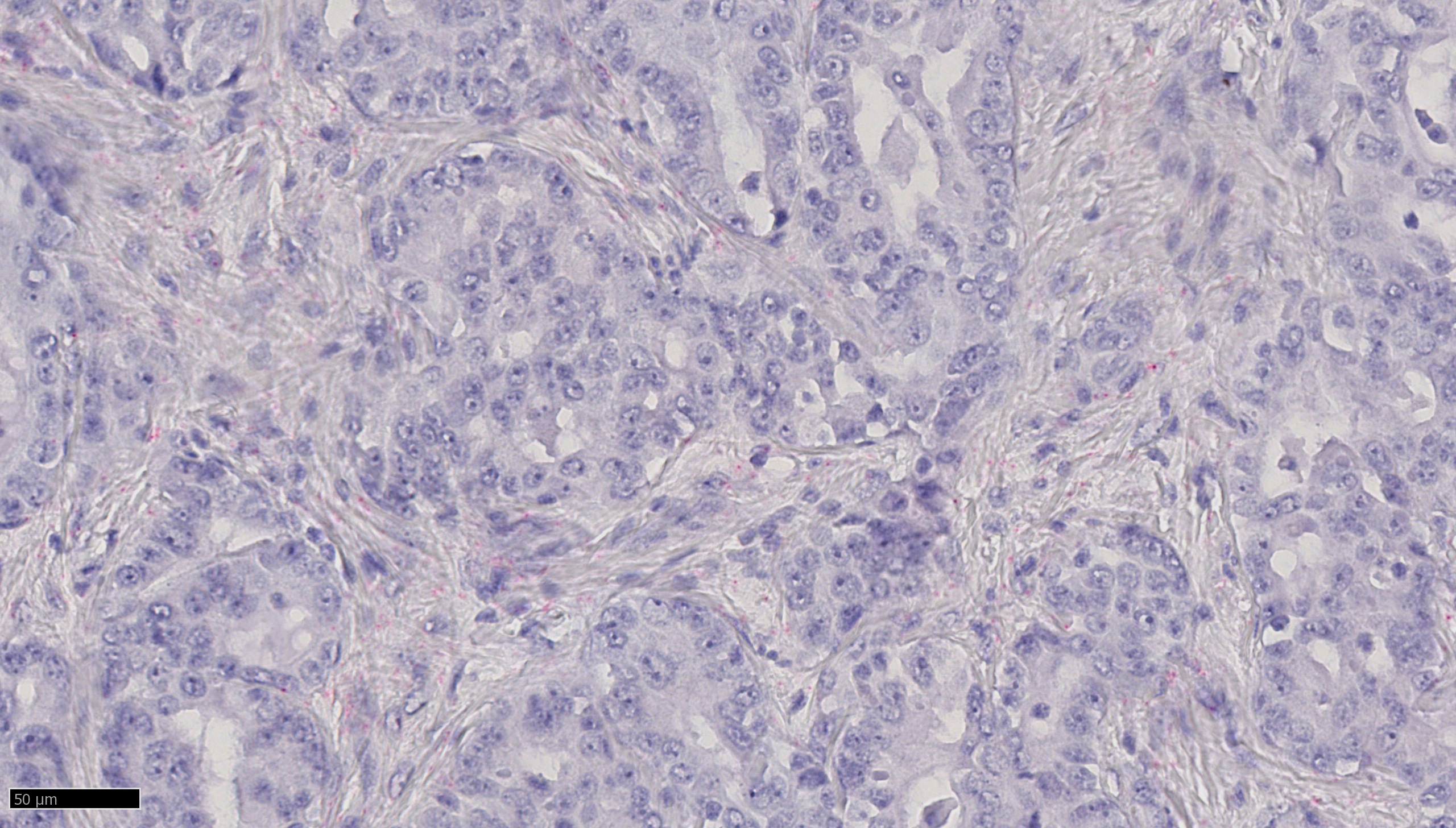

Supplement: S1 File — (ZIP) [file pgen.1011726.s002.zip › S2 figures - Kopi/Bile_duct_2.tif]

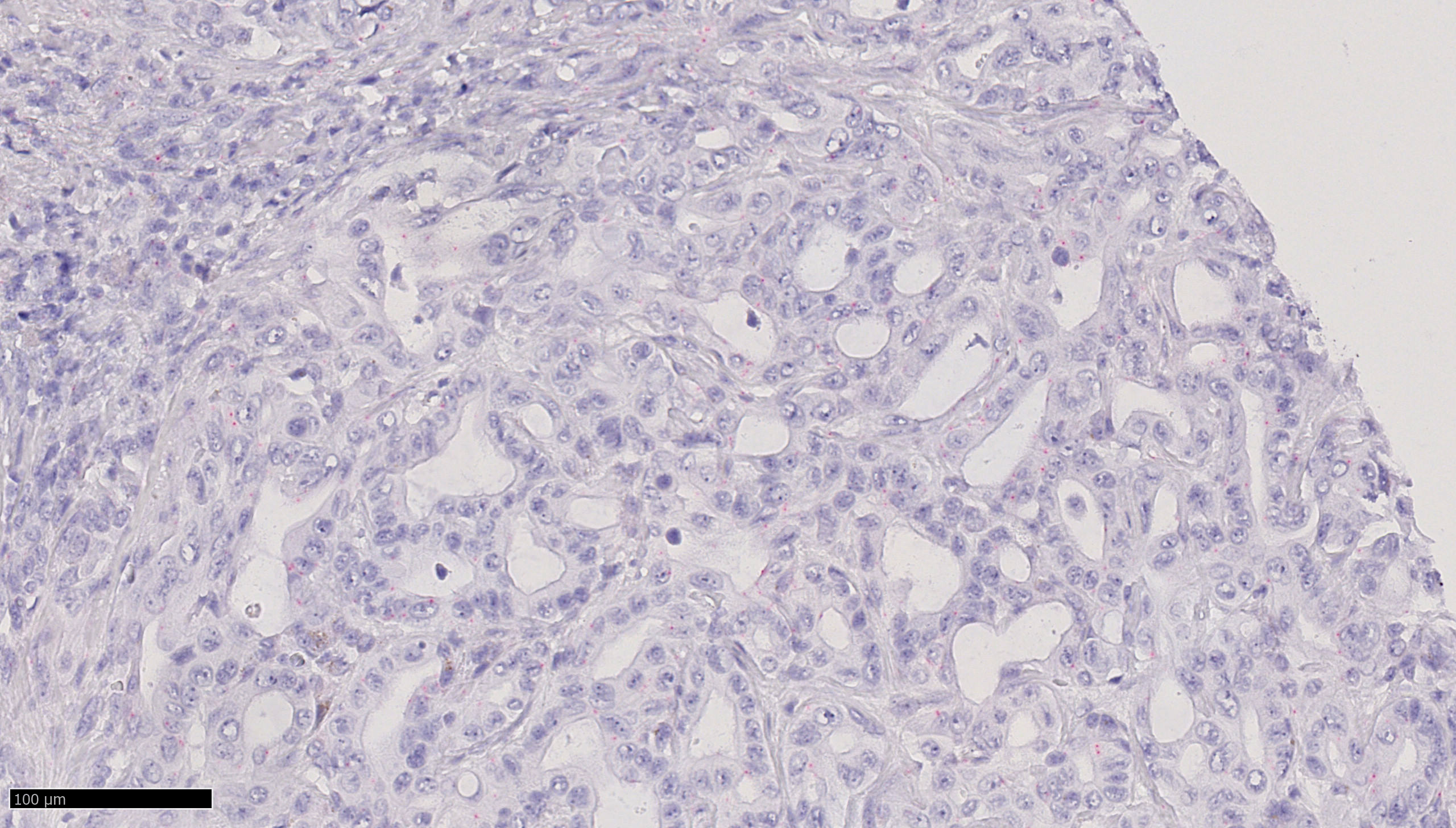

Supplement: S1 File — (ZIP) [file pgen.1011726.s002.zip › S2 figures - Kopi/Bile_duct_3.tif]

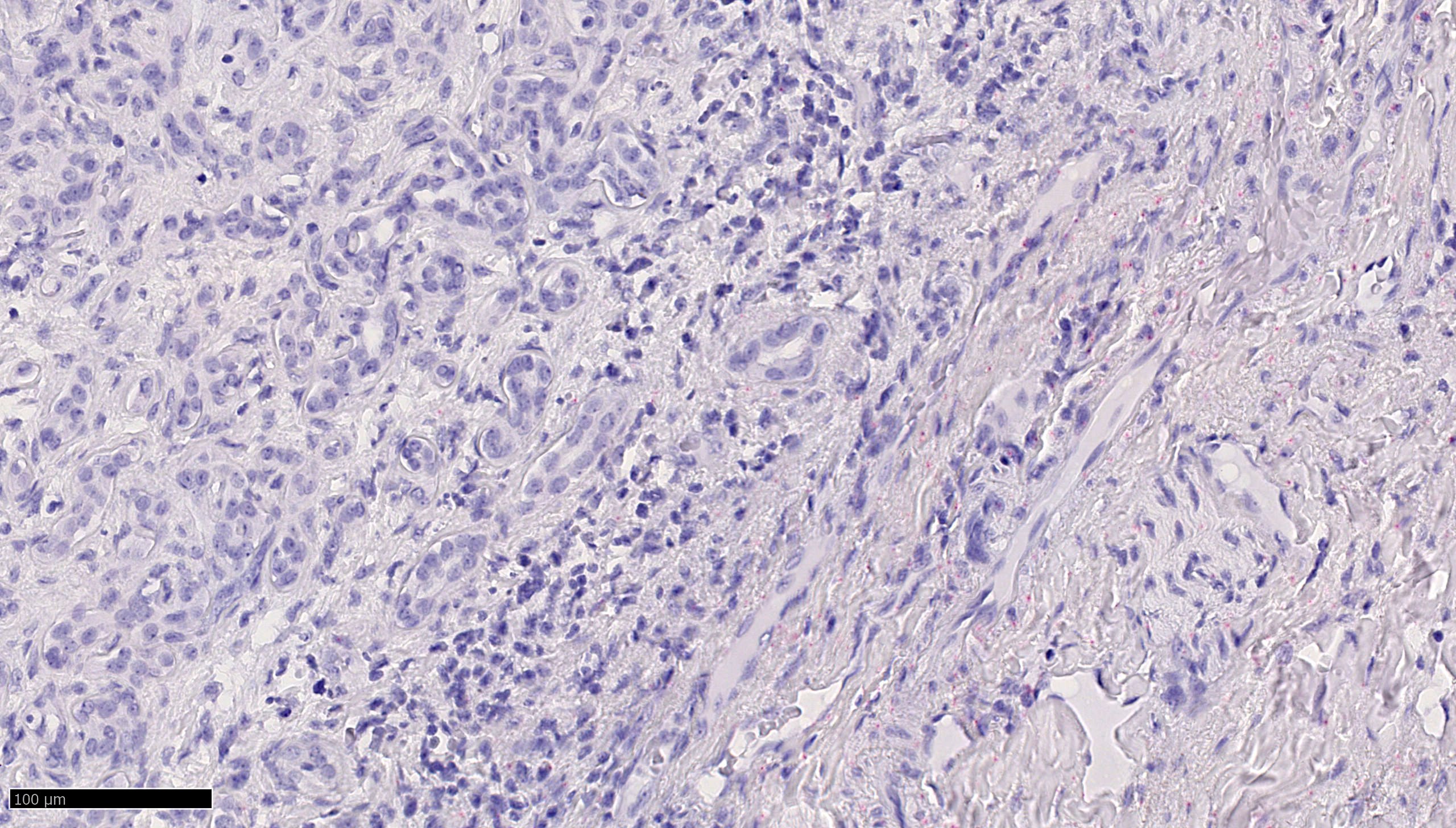

Supplement: S1 File — (ZIP) [file pgen.1011726.s002.zip › S2 figures - Kopi/Bile_duct_4.jpg]

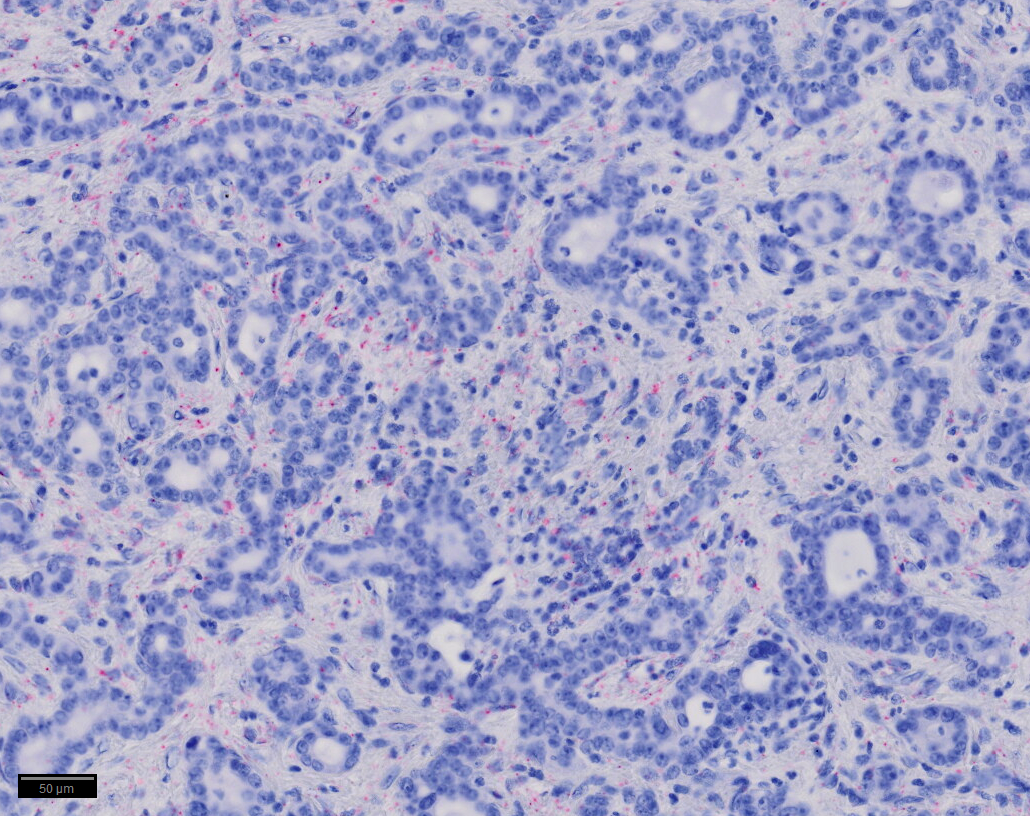

Supplement: S1 File — (ZIP) [file pgen.1011726.s002.zip › S2 figures - Kopi/Bile_duct_5.tif]

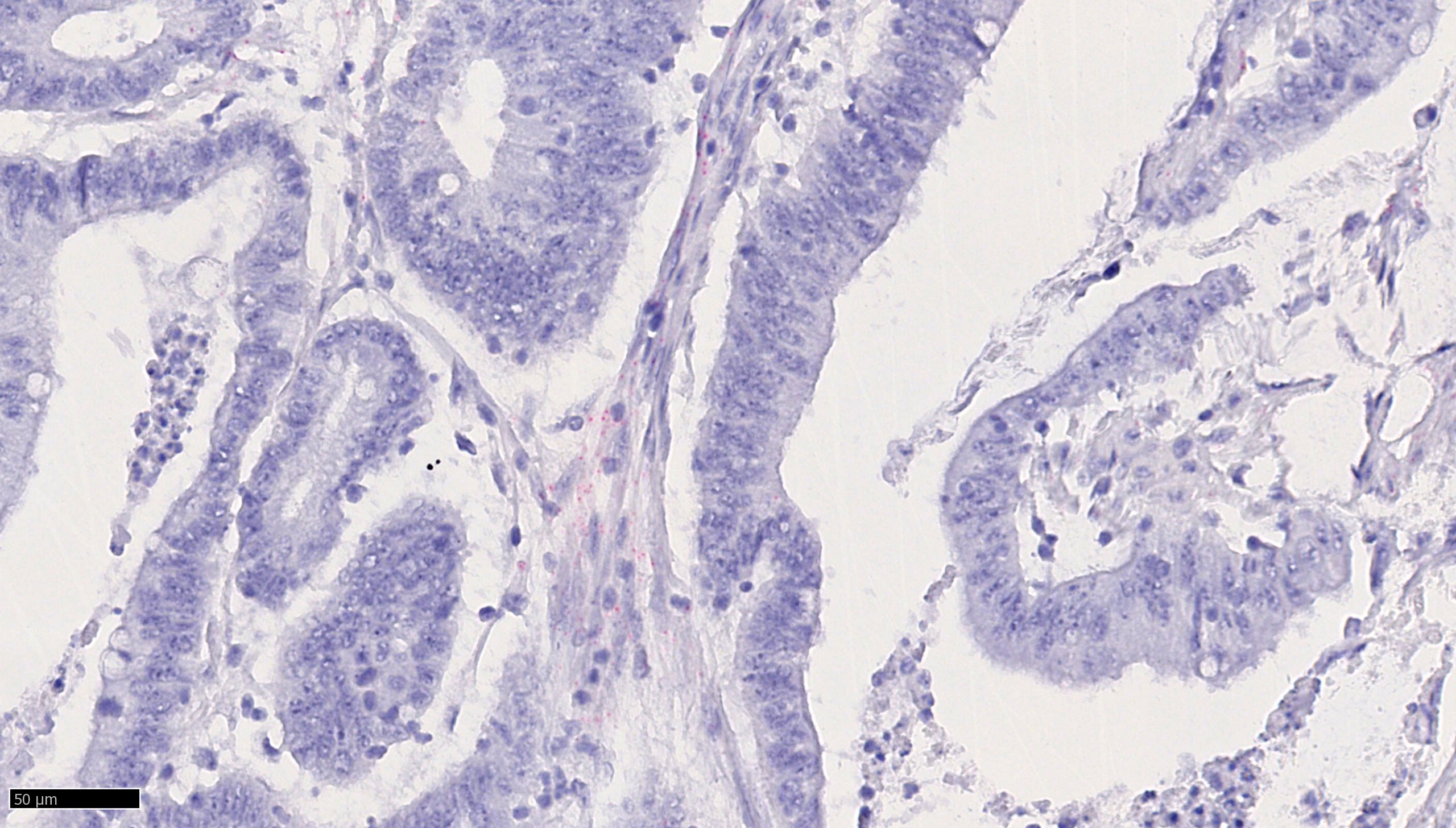

Supplement: S1 File — (ZIP) [file pgen.1011726.s002.zip › S2 figures - Kopi/Colon_1.jpg]

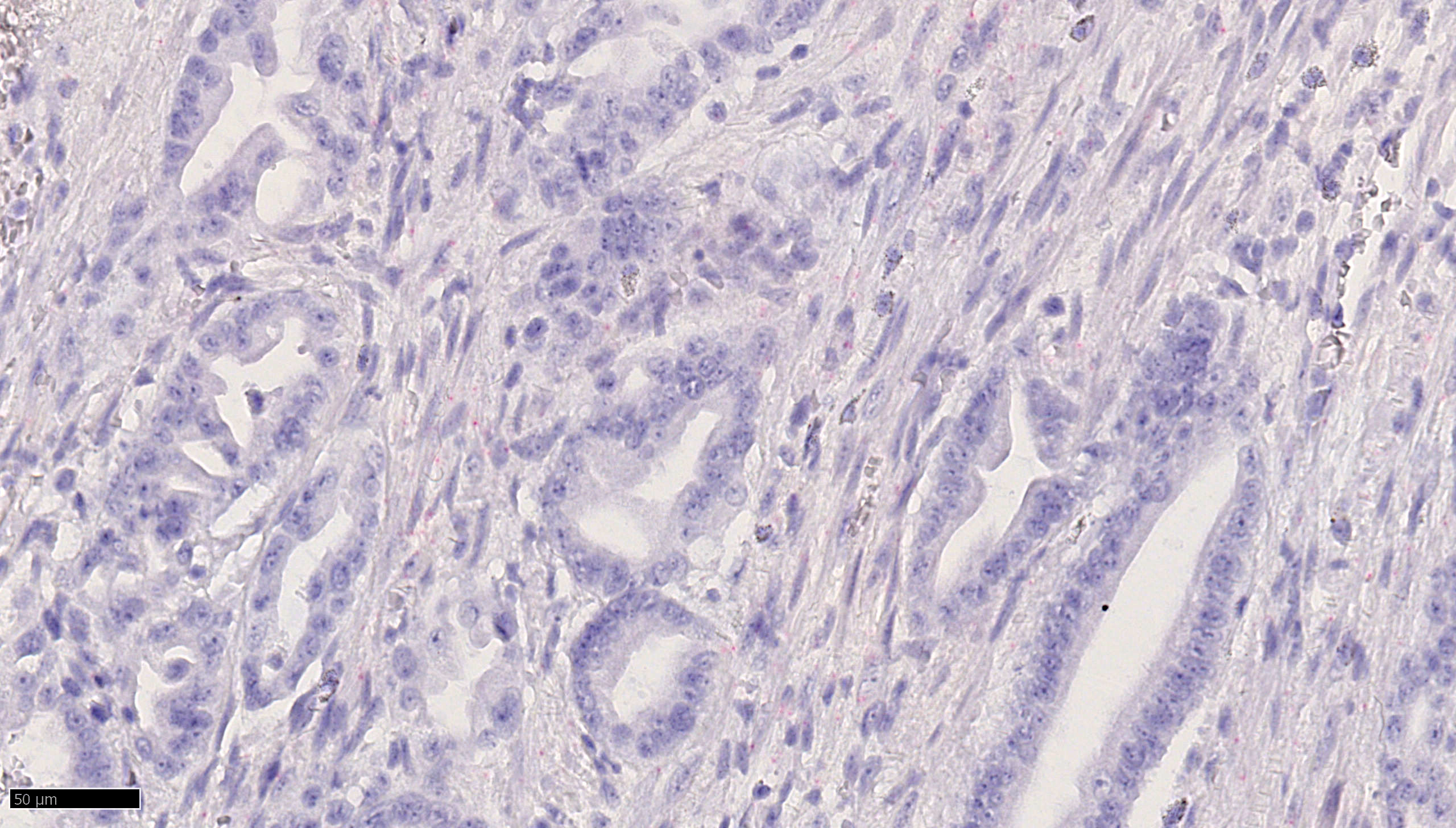

Supplement: S1 File — (ZIP) [file pgen.1011726.s002.zip › S2 figures - Kopi/Colon_2.tif]

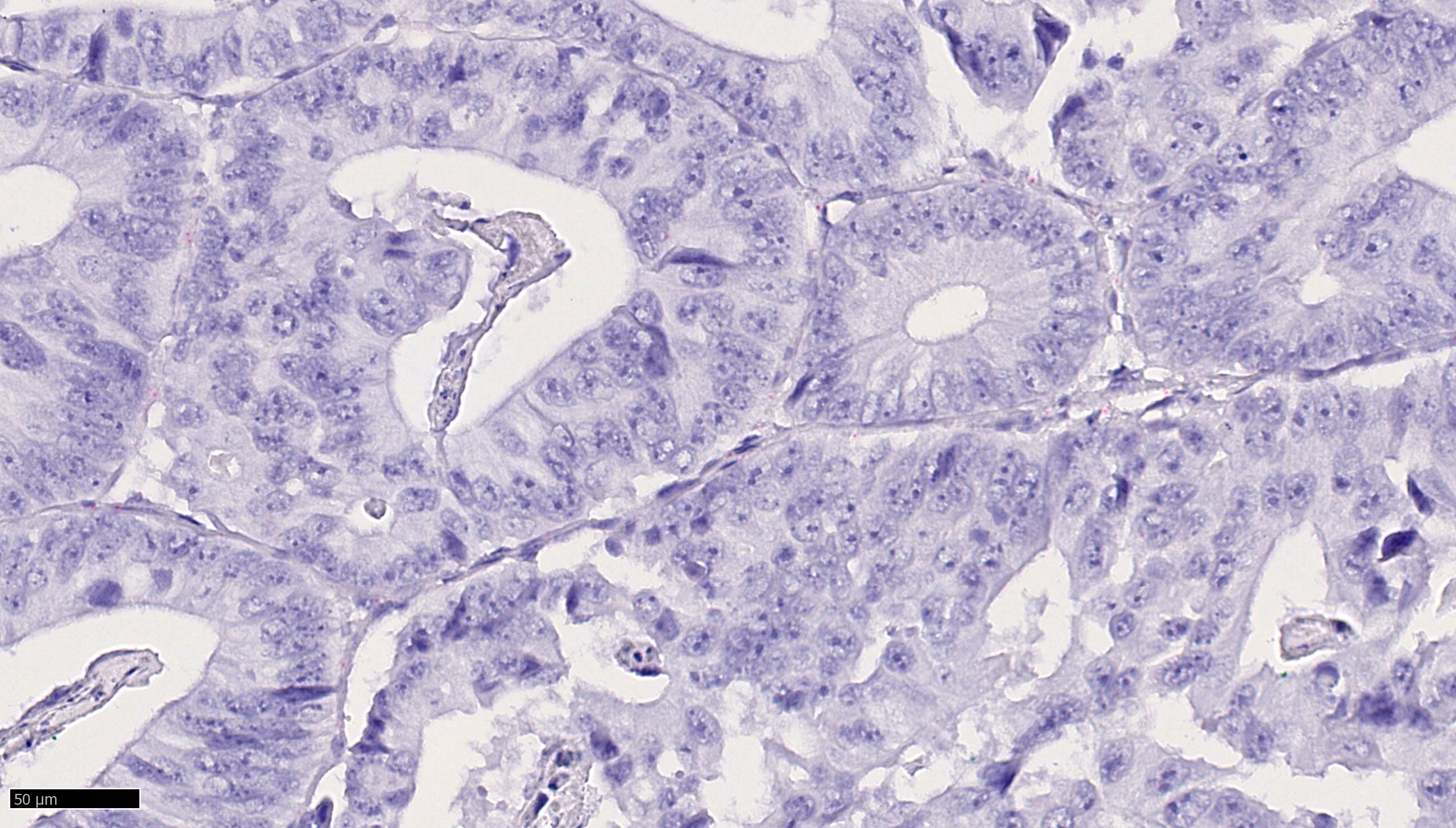

Supplement: S1 File — (ZIP) [file pgen.1011726.s002.zip › S2 figures - Kopi/Colon_3.jpg]

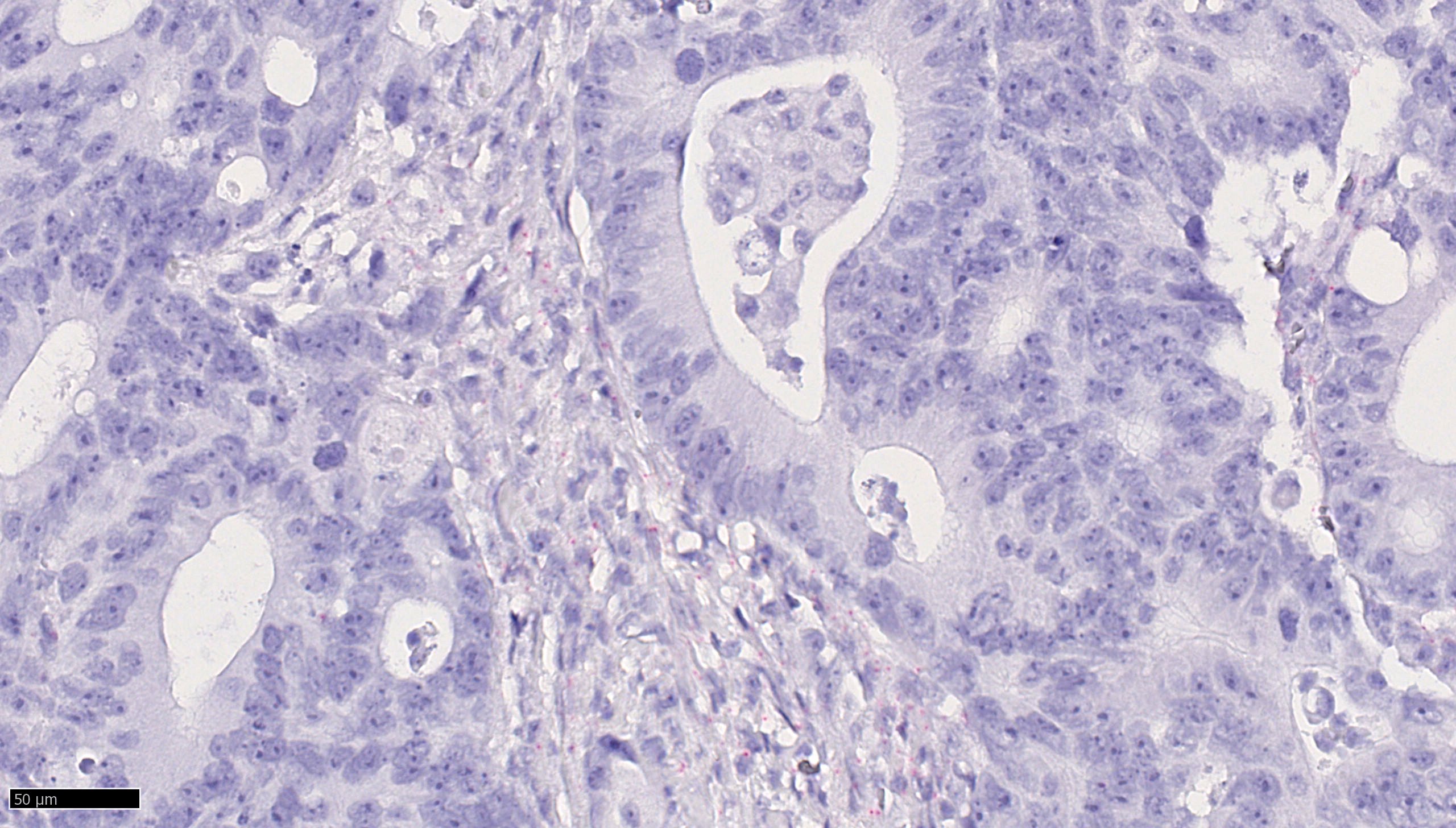

Supplement: S1 File — (ZIP) [file pgen.1011726.s002.zip › S2 figures - Kopi/Colon_4.jpg]

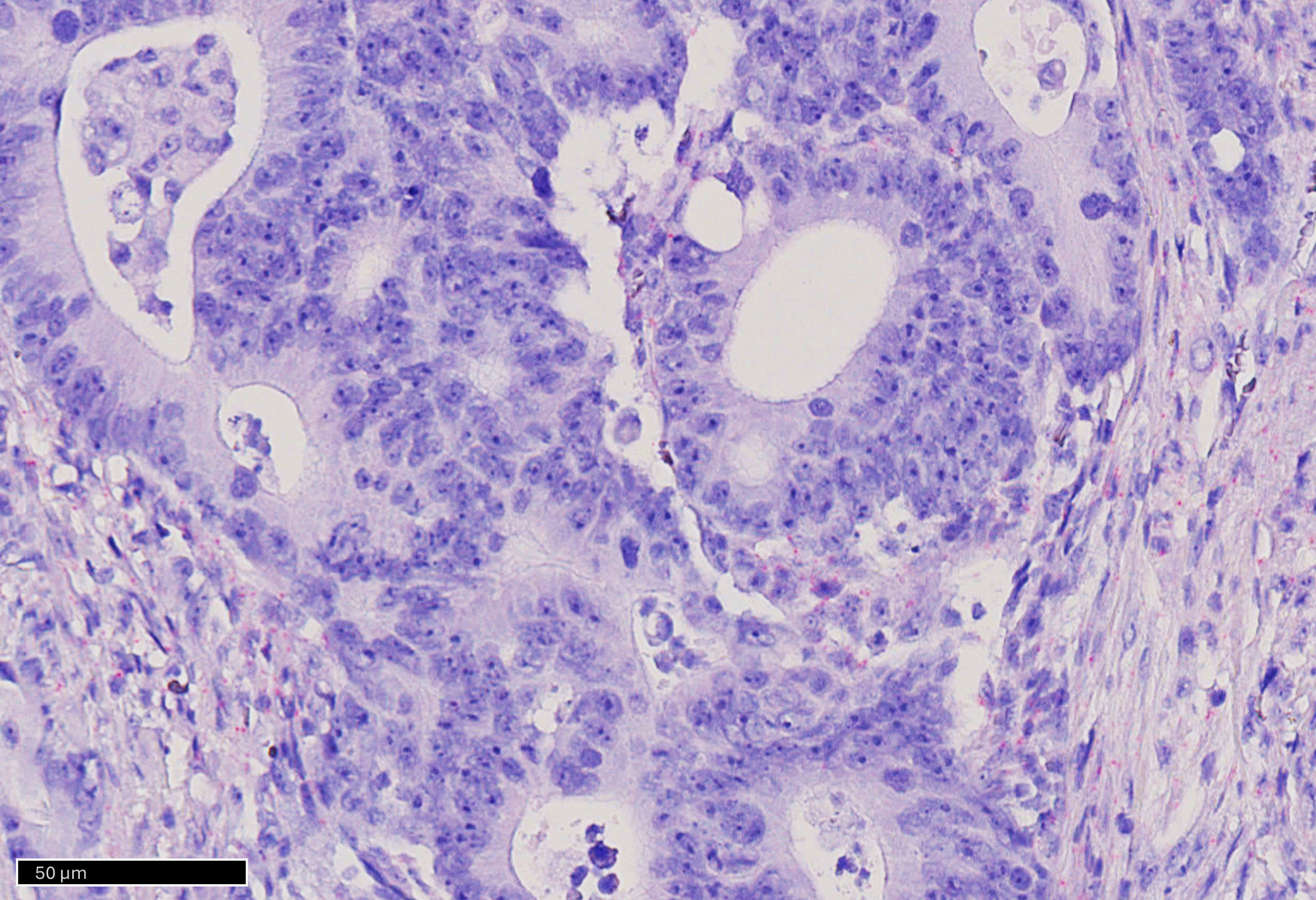

Supplement: S1 File — (ZIP) [file pgen.1011726.s002.zip › S2 figures - Kopi/Colon_5.tif]

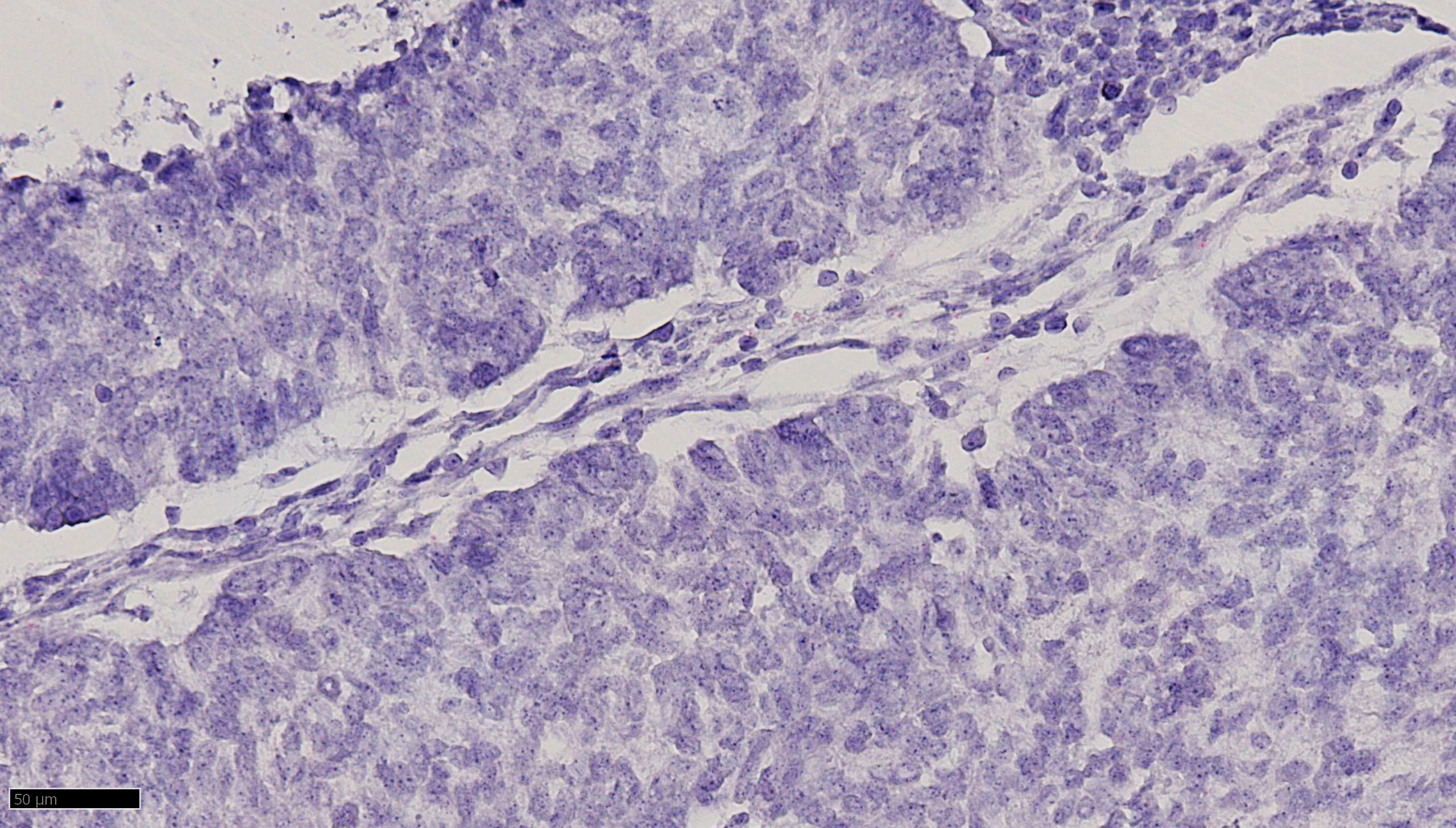

Supplement: S1 File — (ZIP) [file pgen.1011726.s002.zip › S2 figures - Kopi/Endometrium_1.jpg]

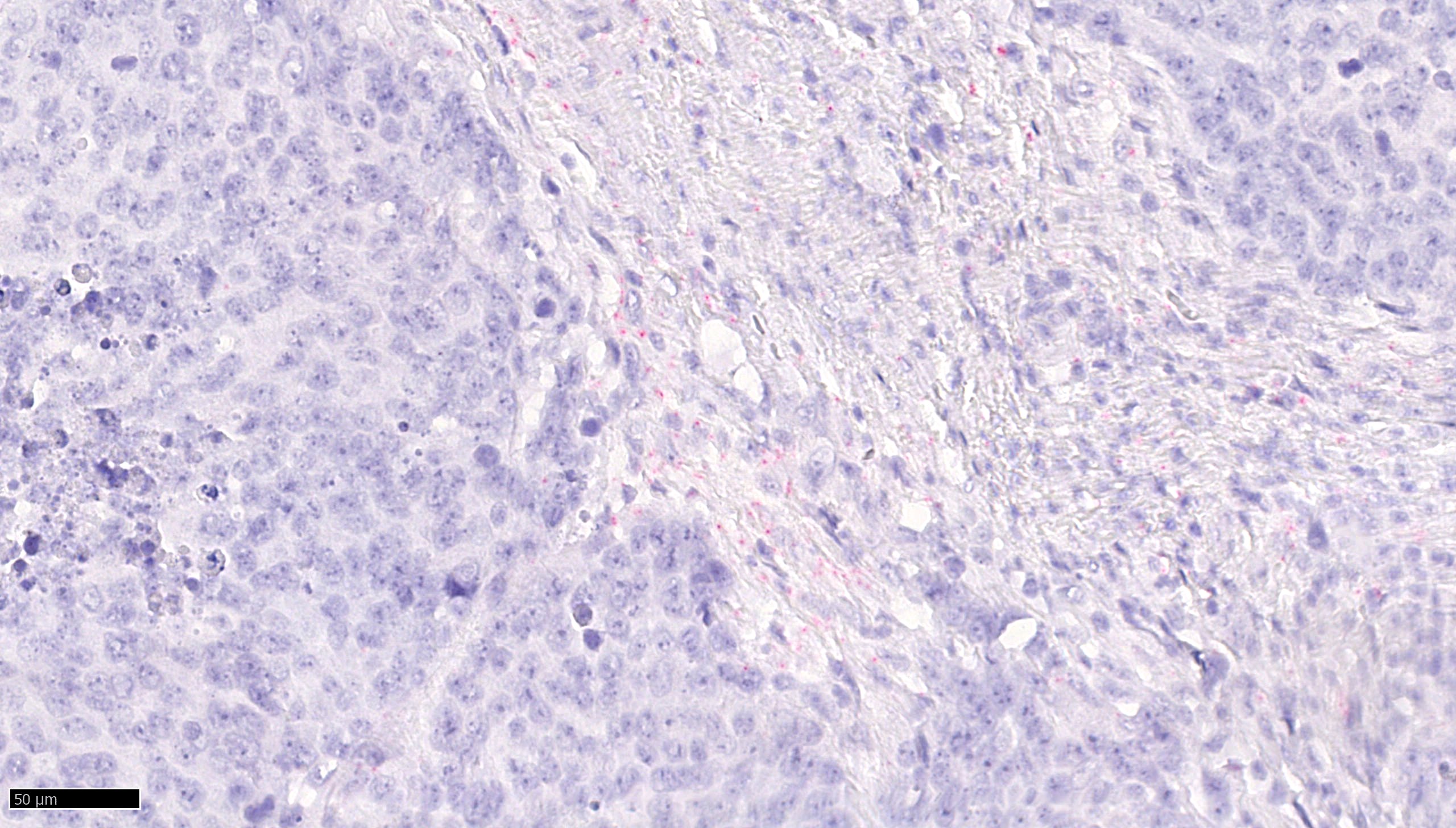

Supplement: S1 File — (ZIP) [file pgen.1011726.s002.zip › S2 figures - Kopi/Endometrium_2.jpg]

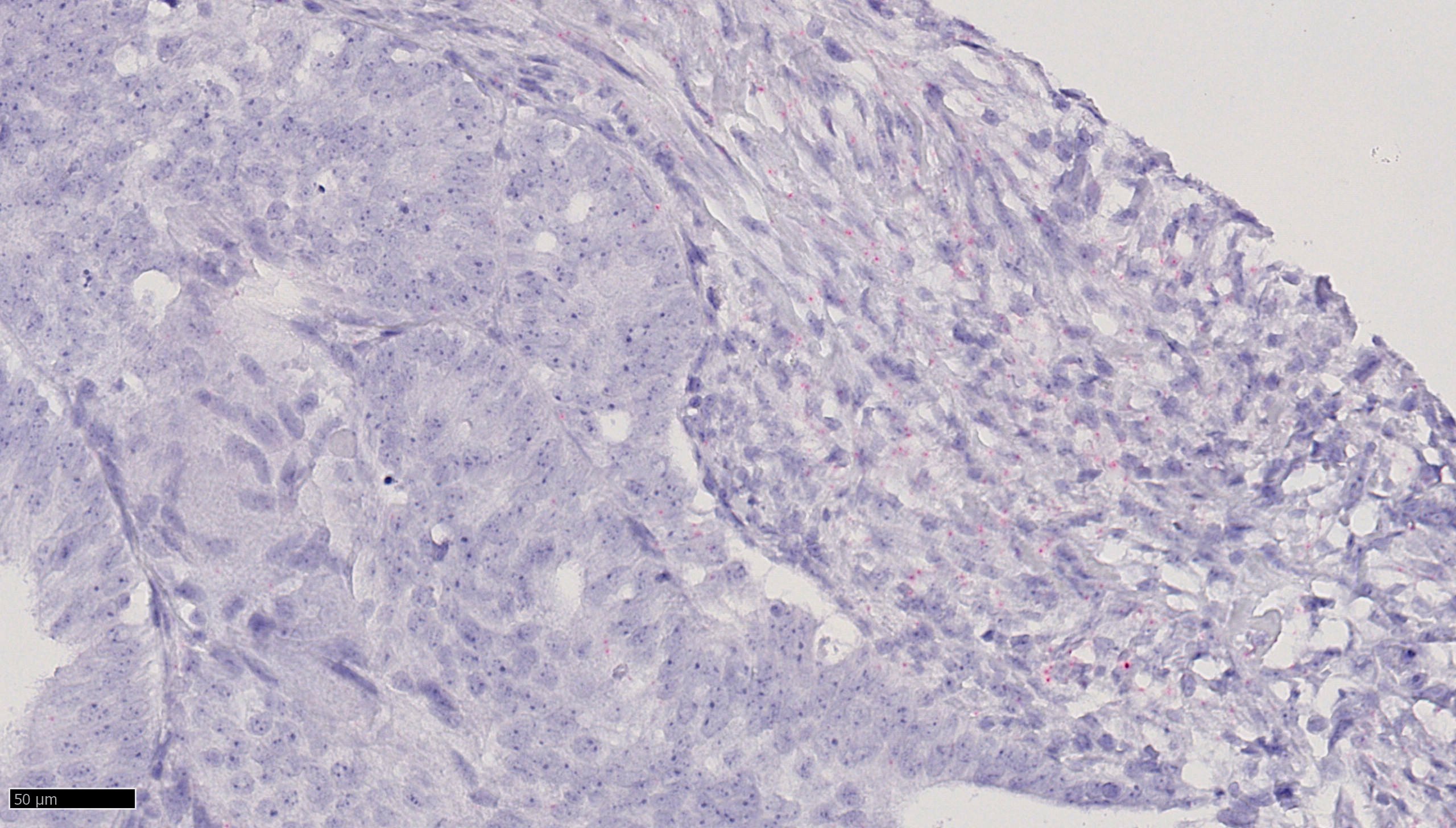

Supplement: S1 File — (ZIP) [file pgen.1011726.s002.zip › S2 figures - Kopi/Endometrium_3.jpg]

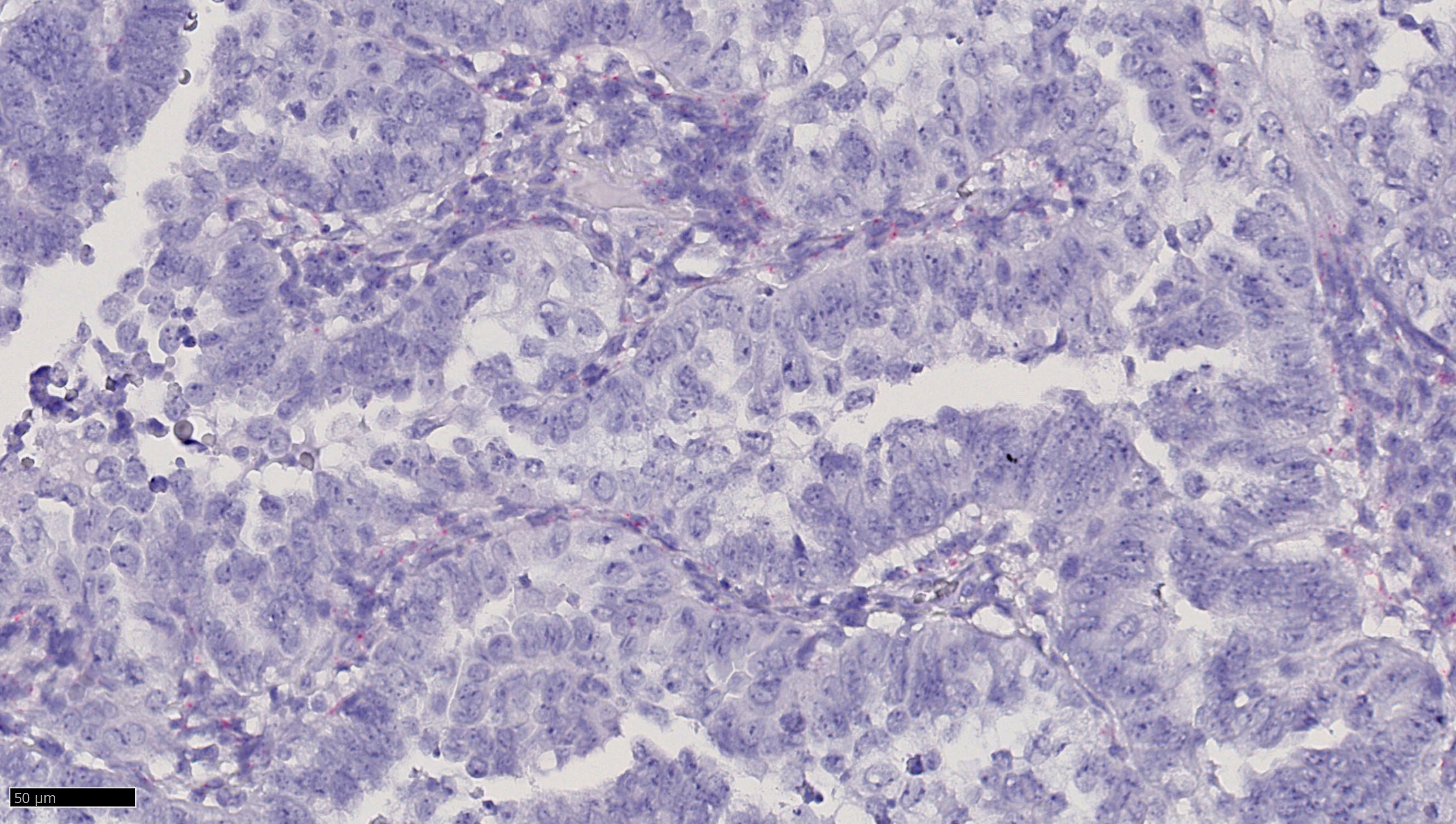

Supplement: S1 File — (ZIP) [file pgen.1011726.s002.zip › S2 figures - Kopi/Endometrium_4.jpg]

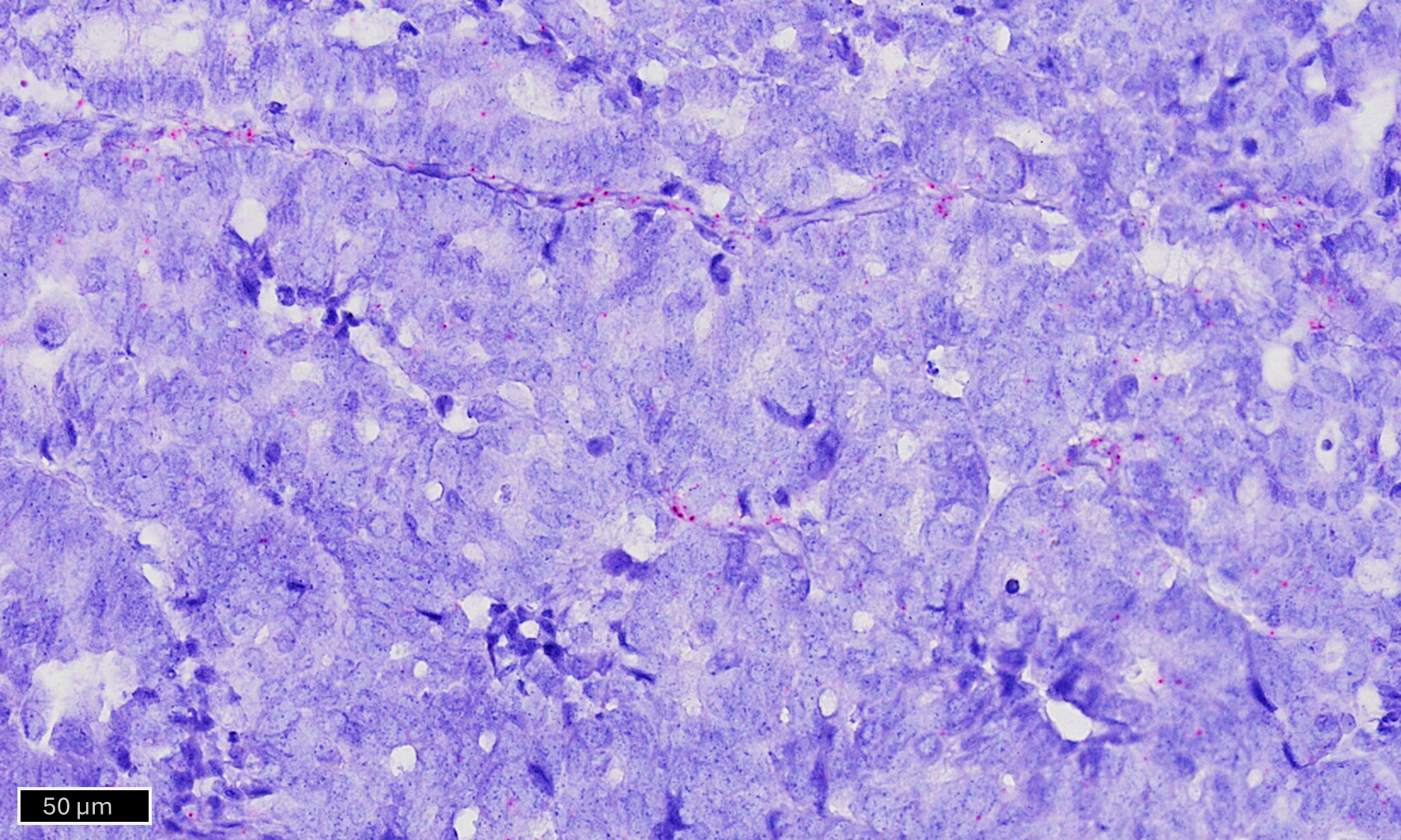

Supplement: S1 File — (ZIP) [file pgen.1011726.s002.zip › S2 figures - Kopi/Endometrium_5.tif]

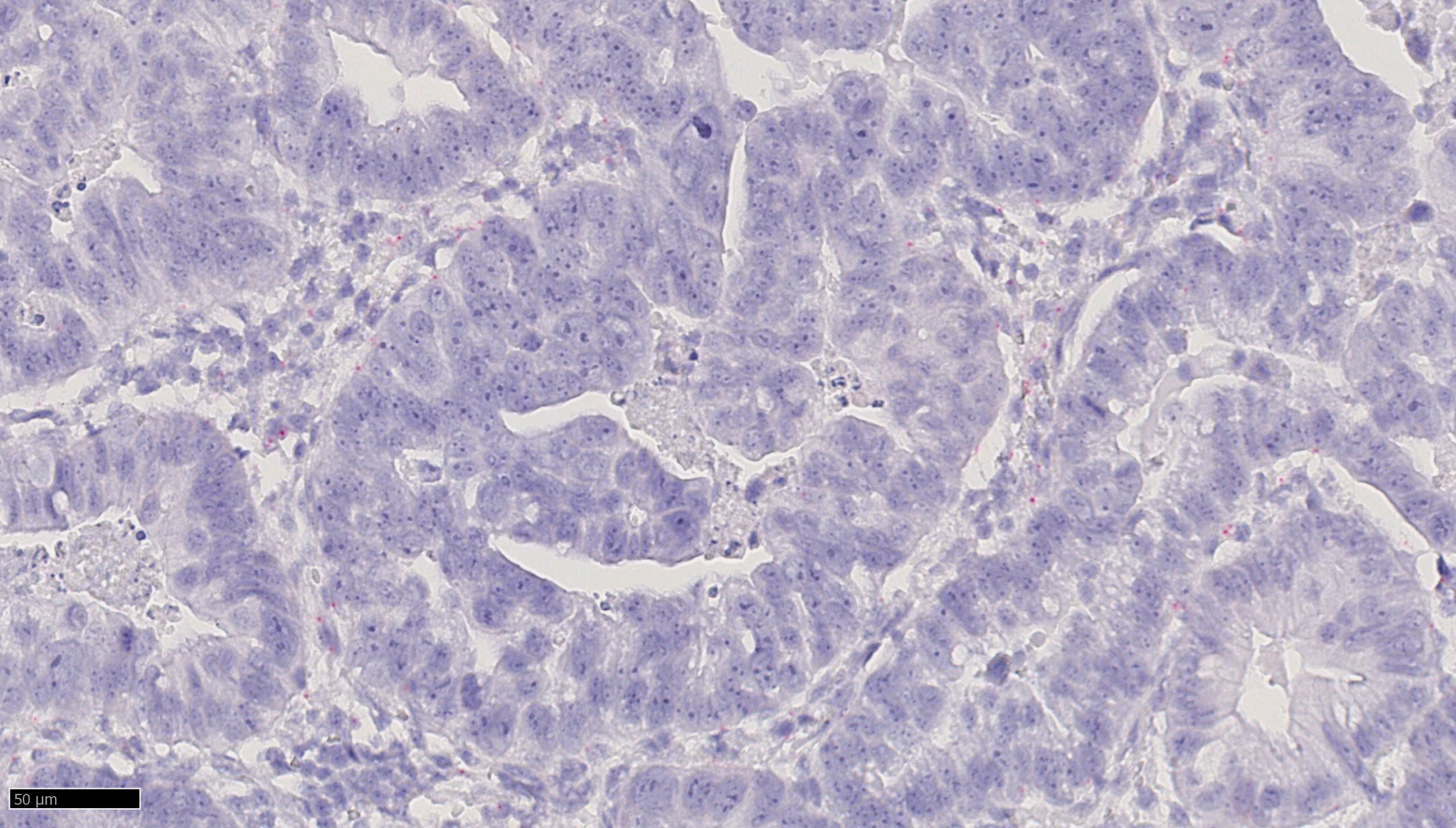

Supplement: S1 File — (ZIP) [file pgen.1011726.s002.zip › S2 figures - Kopi/GEJ_1.jpg]

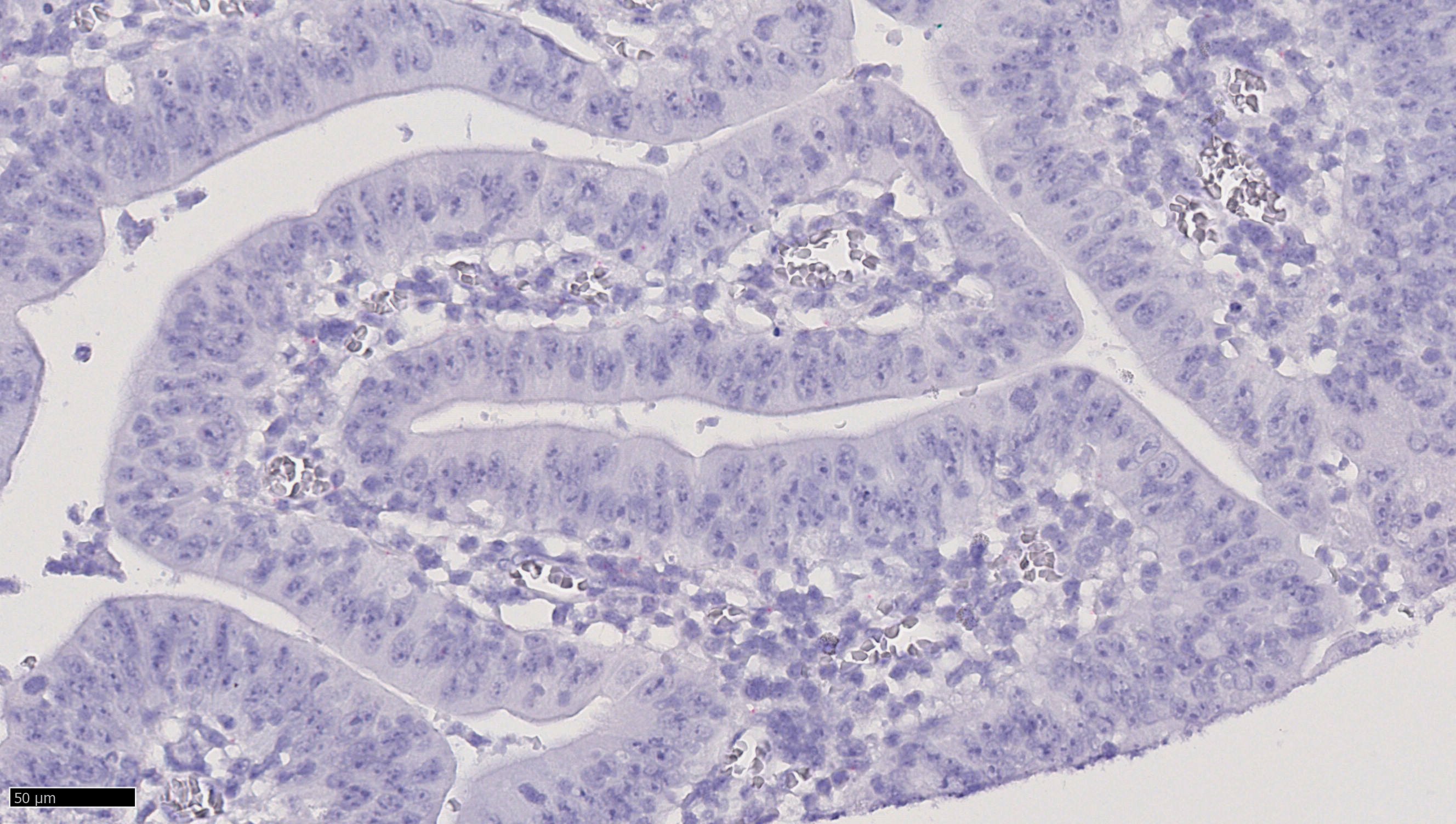

Supplement: S1 File — (ZIP) [file pgen.1011726.s002.zip › S2 figures - Kopi/GEJ_2.jpg]

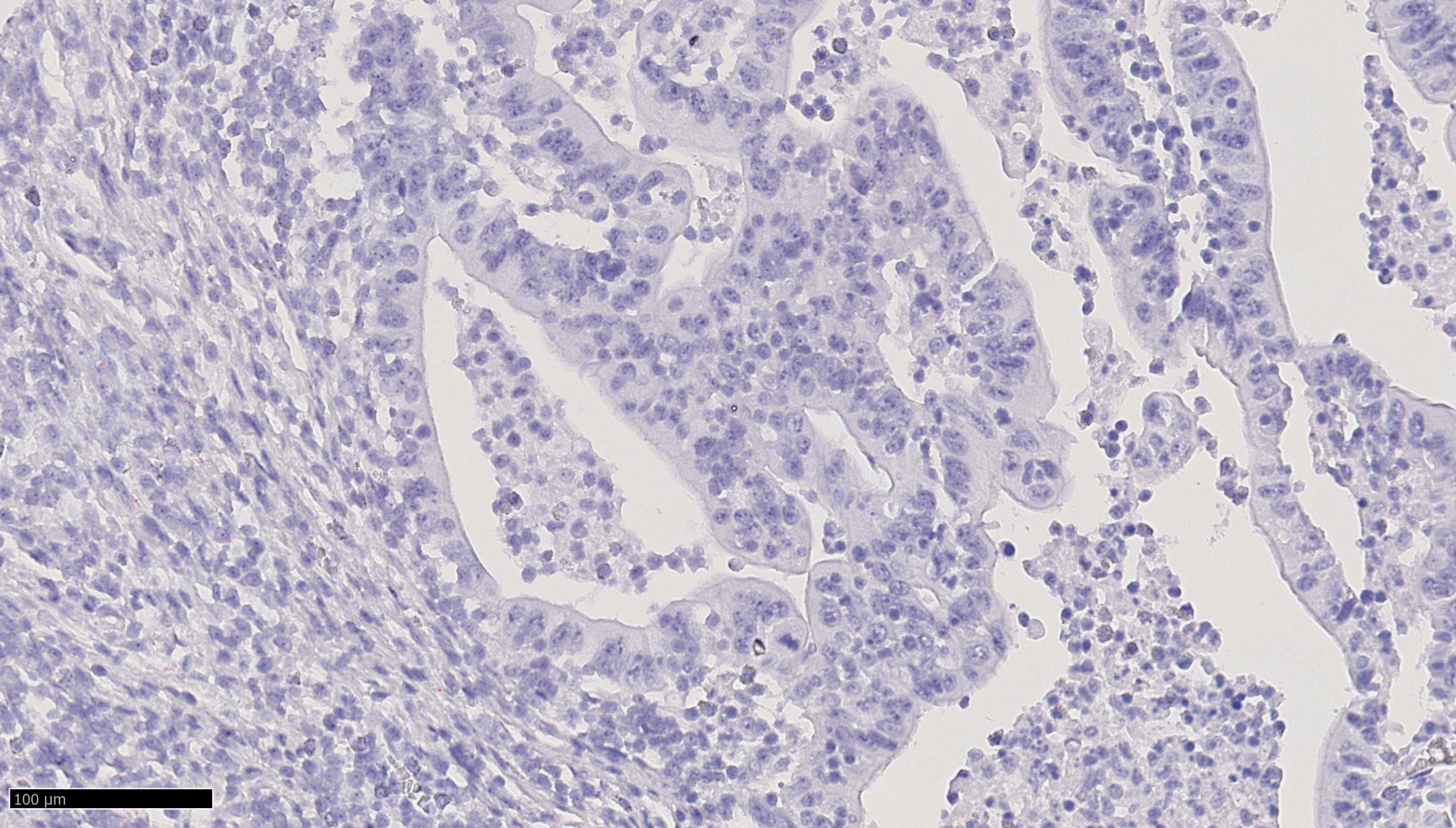

Supplement: S1 File — (ZIP) [file pgen.1011726.s002.zip › S2 figures - Kopi/GEJ_3.jpg]

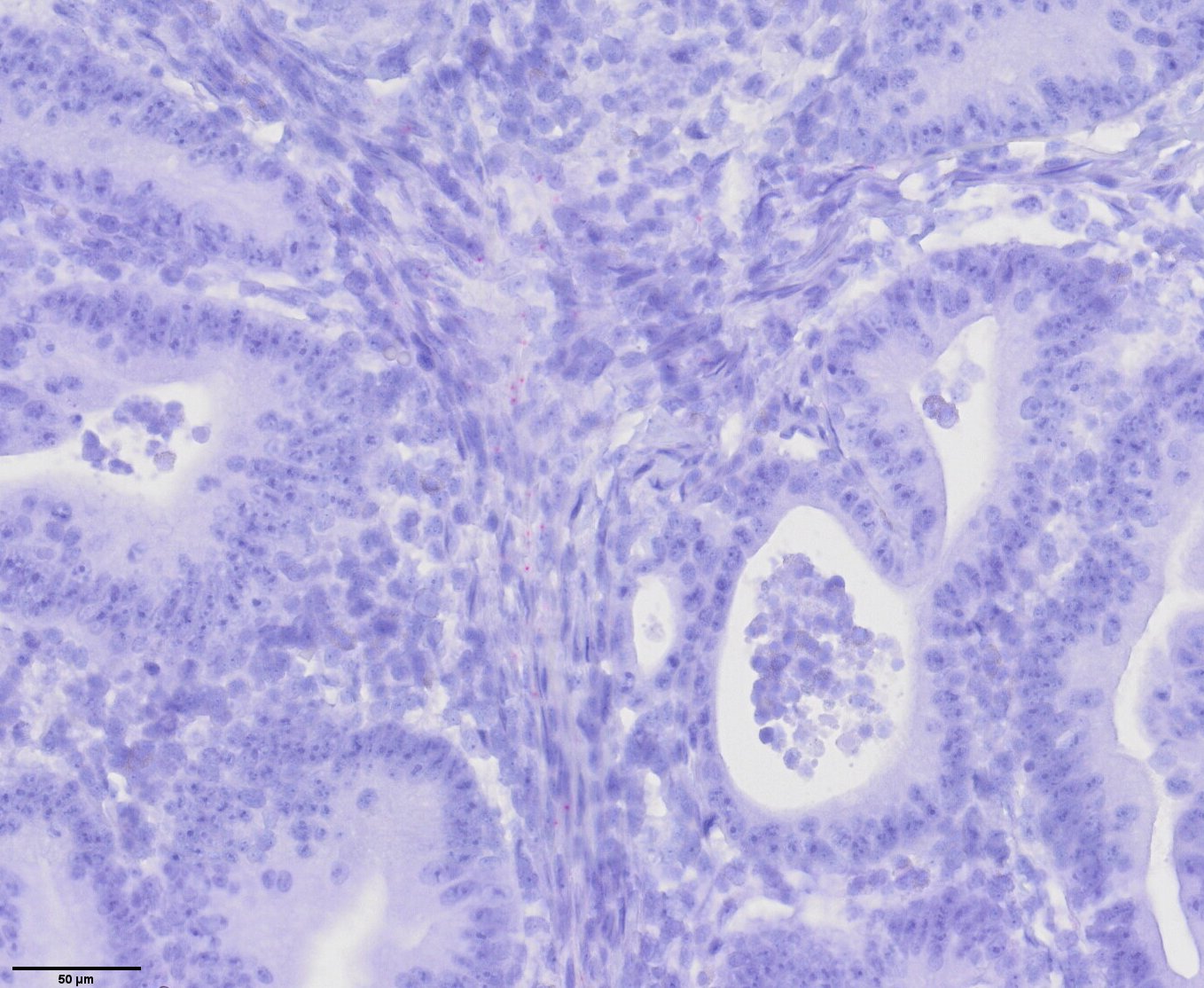

Supplement: S1 File — (ZIP) [file pgen.1011726.s002.zip › S2 figures - Kopi/GEJ_4.jpg]

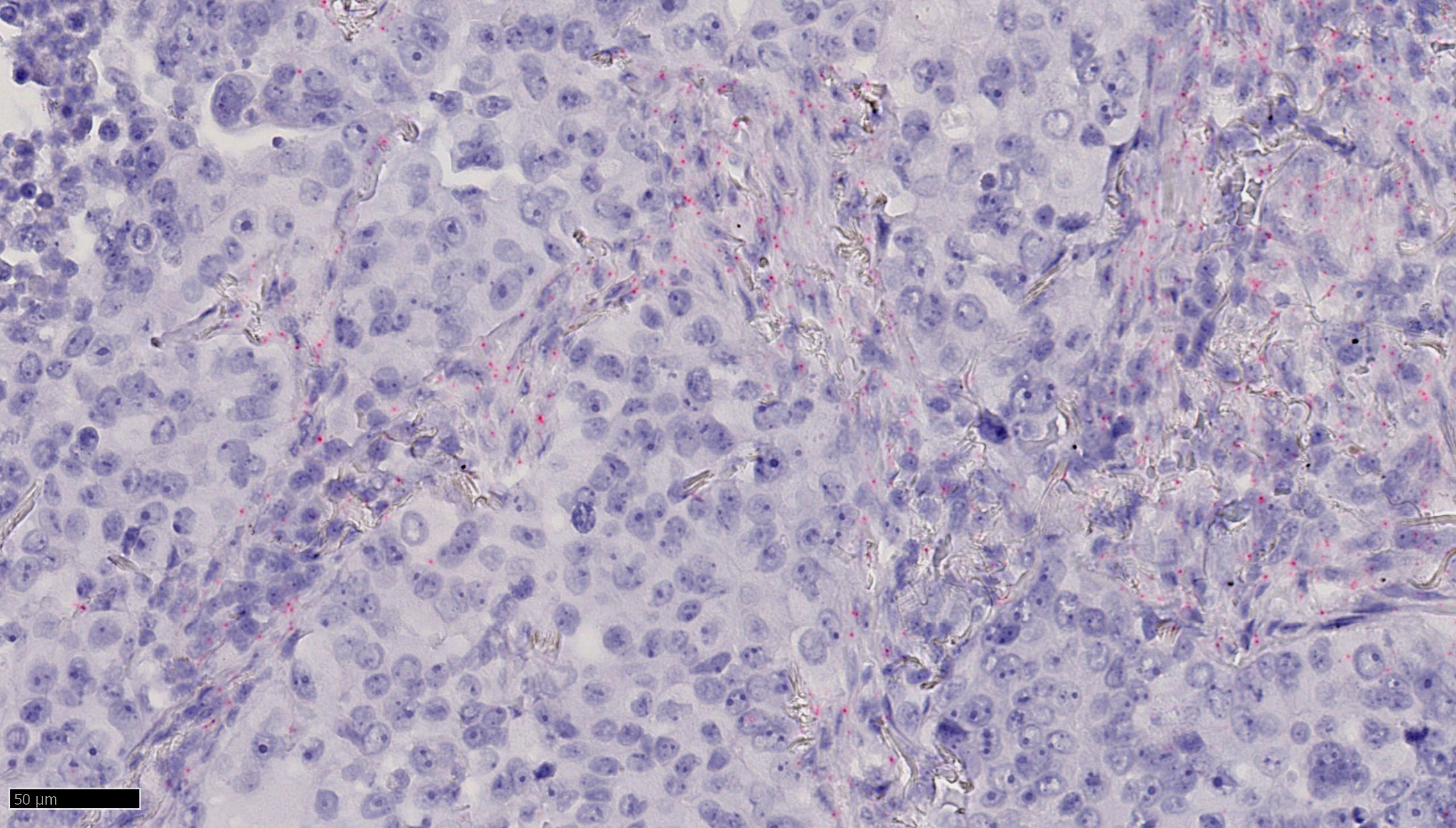

Supplement: S1 File — (ZIP) [file pgen.1011726.s002.zip › S2 figures - Kopi/Lung_1.jpg]

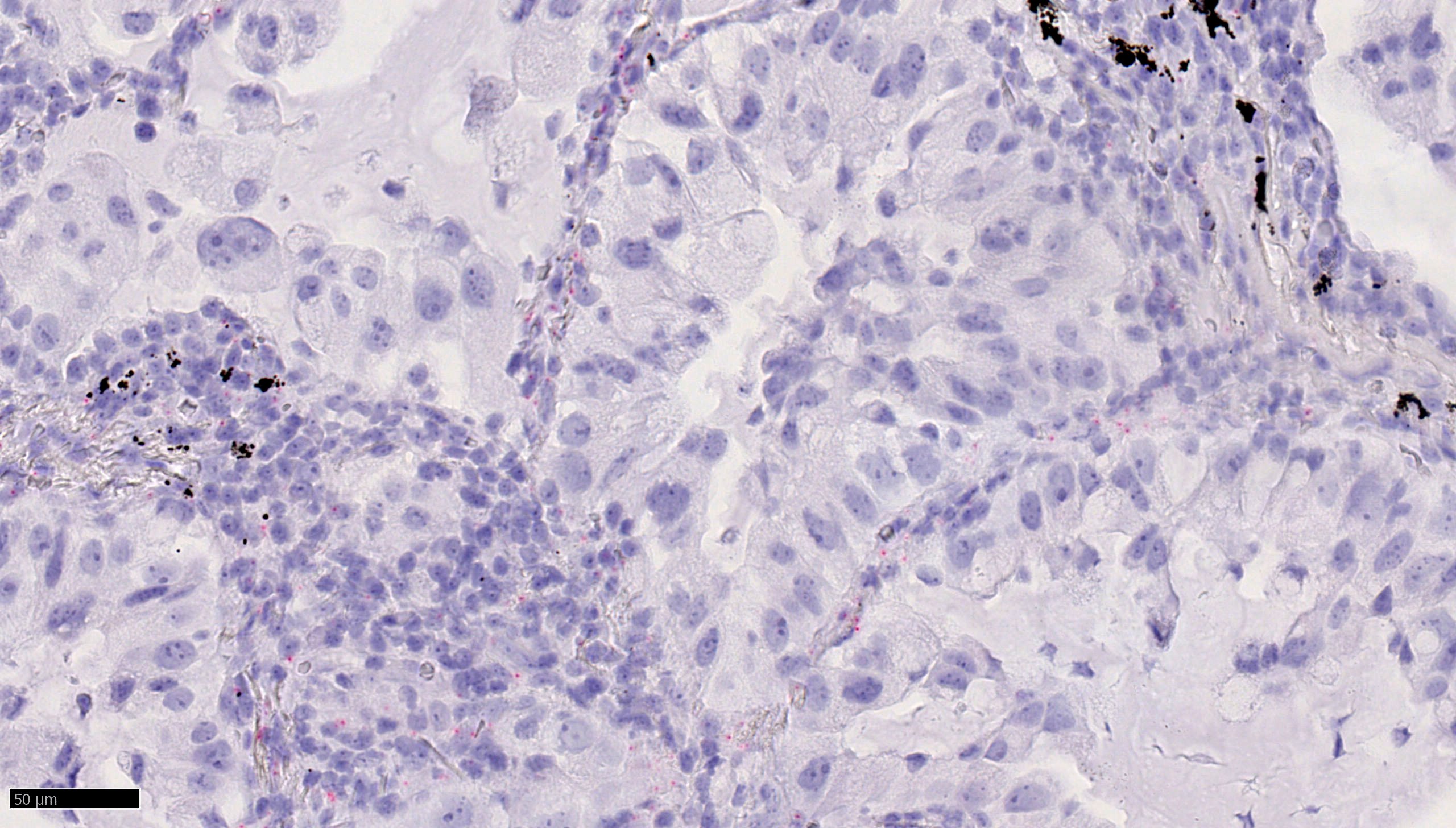

Supplement: S1 File — (ZIP) [file pgen.1011726.s002.zip › S2 figures - Kopi/Lung_2.jpg]

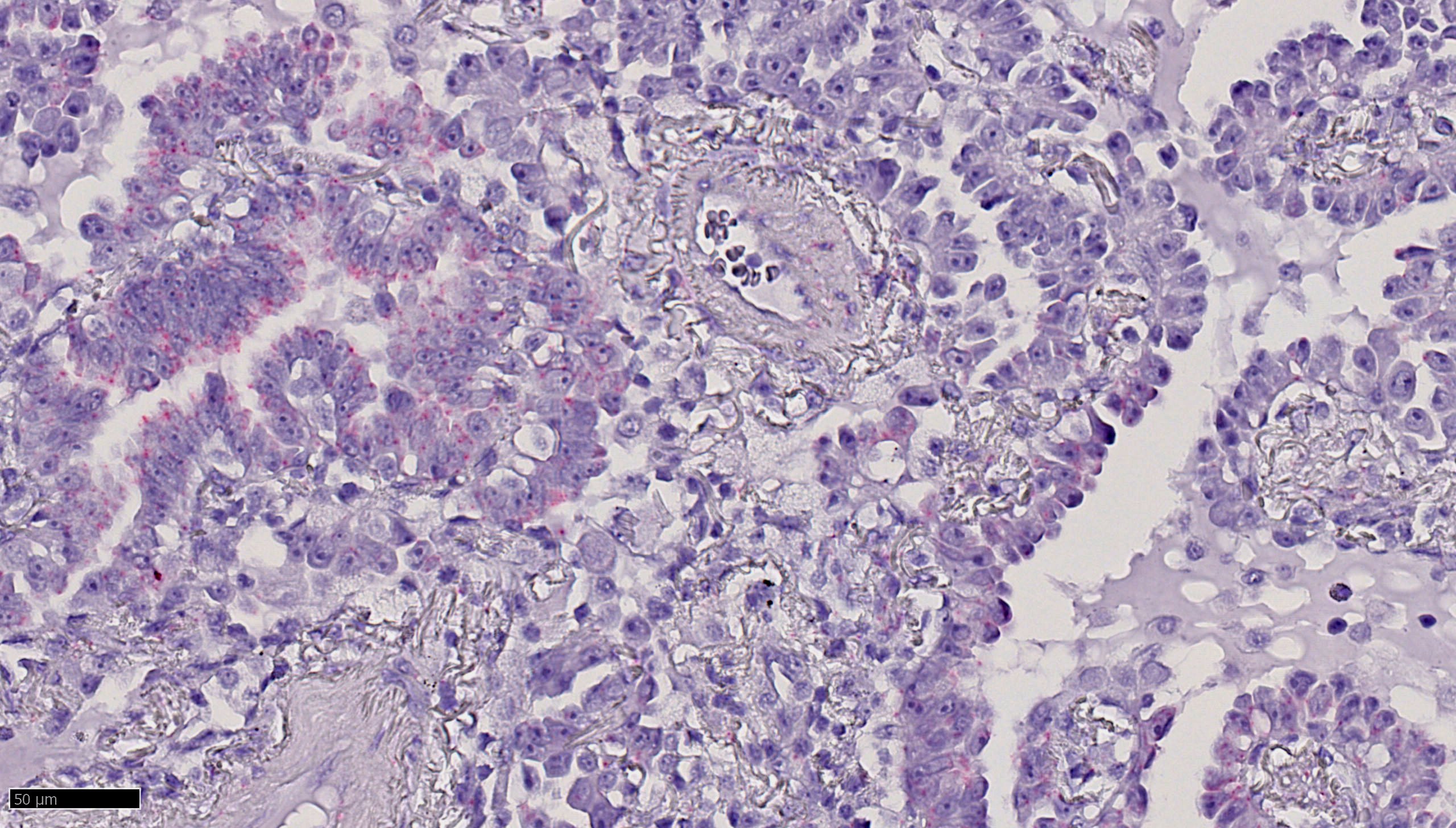

Supplement: S1 File — (ZIP) [file pgen.1011726.s002.zip › S2 figures - Kopi/Lung_3.jpg]

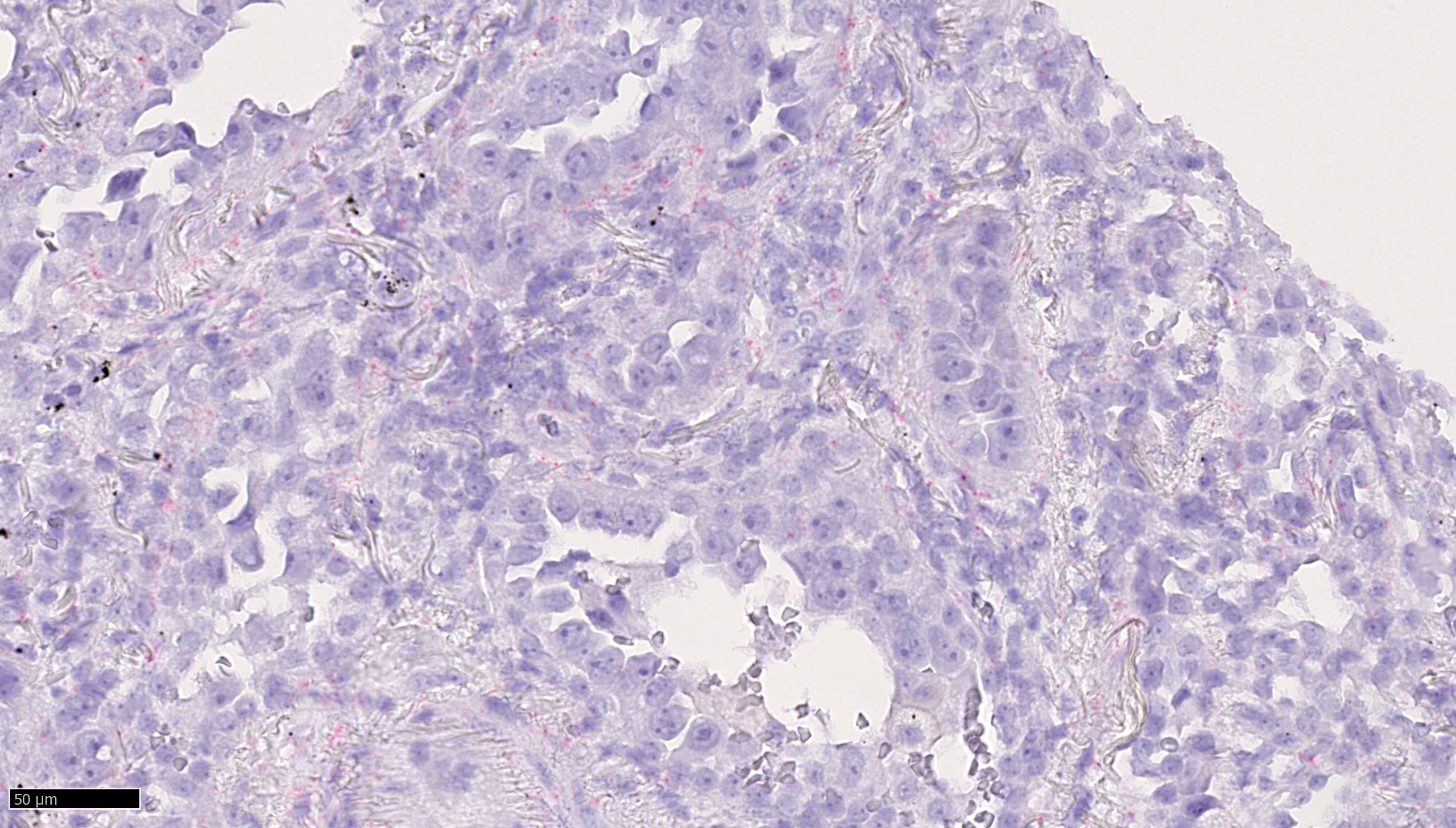

Supplement: S1 File — (ZIP) [file pgen.1011726.s002.zip › S2 figures - Kopi/Lung_4.jpg]

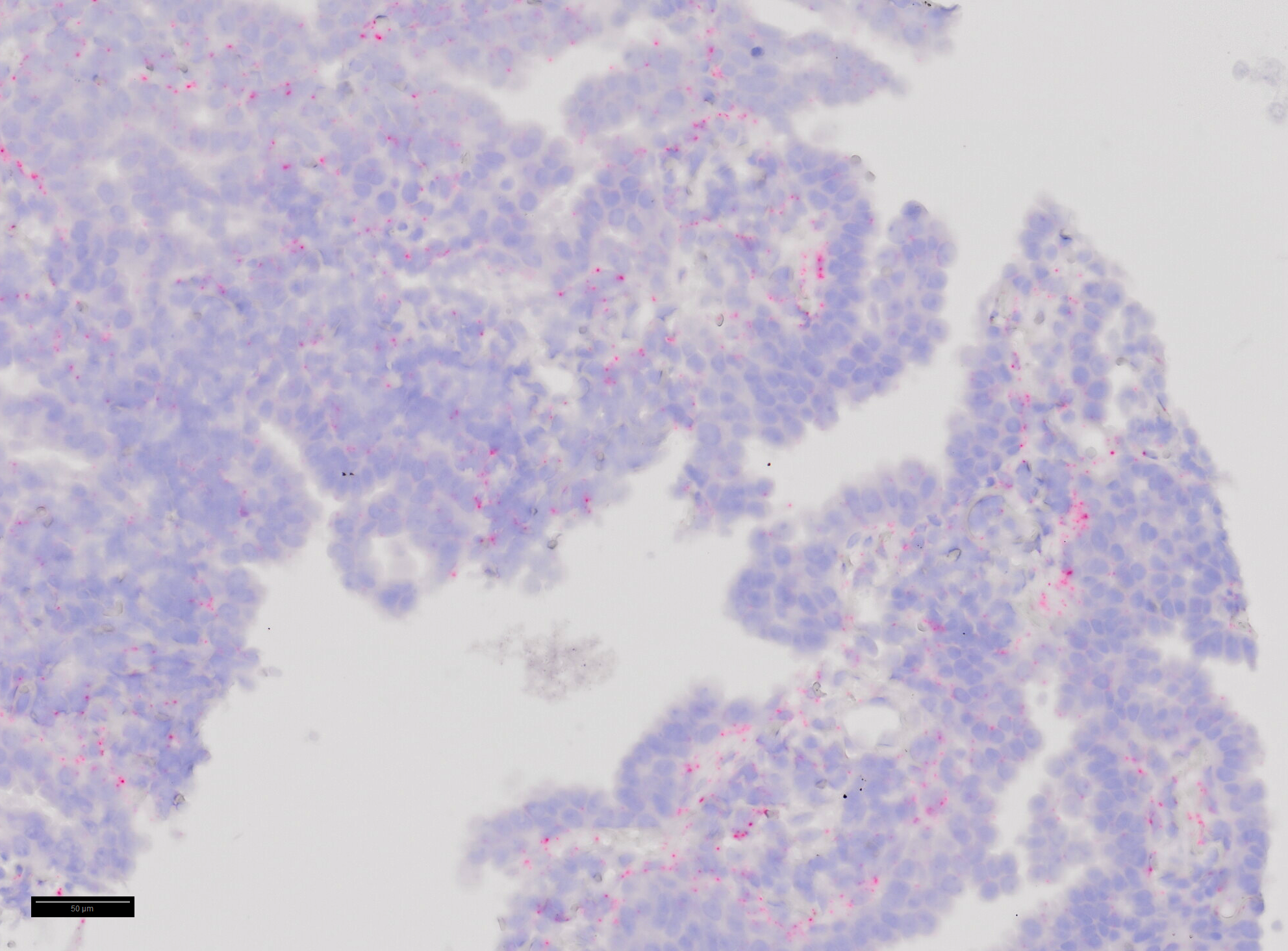

Supplement: S1 File — (ZIP) [file pgen.1011726.s002.zip › S2 figures - Kopi/Lung_5.tif]

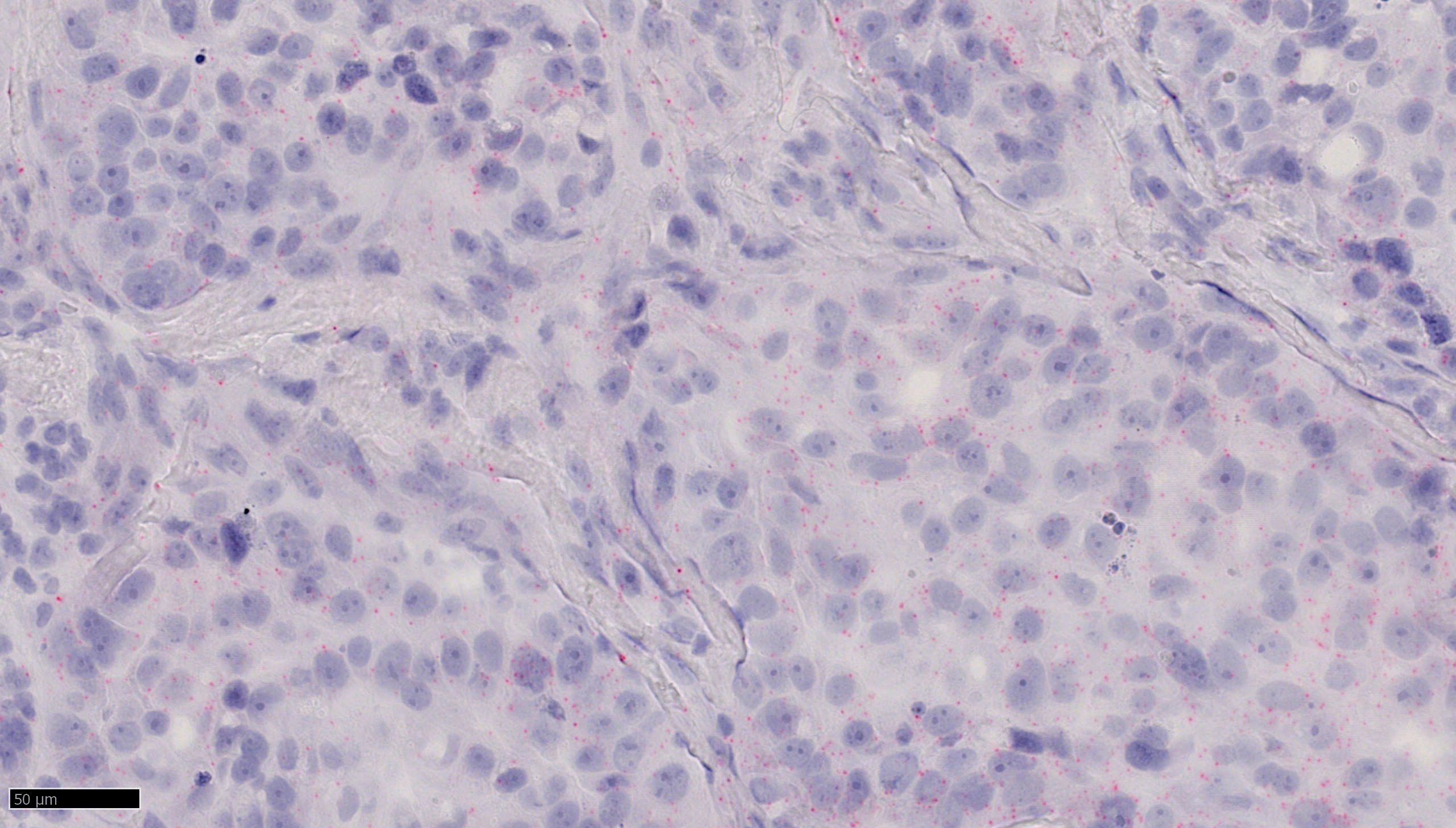

Supplement: S1 File — (ZIP) [file pgen.1011726.s002.zip › S2 figures - Kopi/Mamma pt1.jpg]

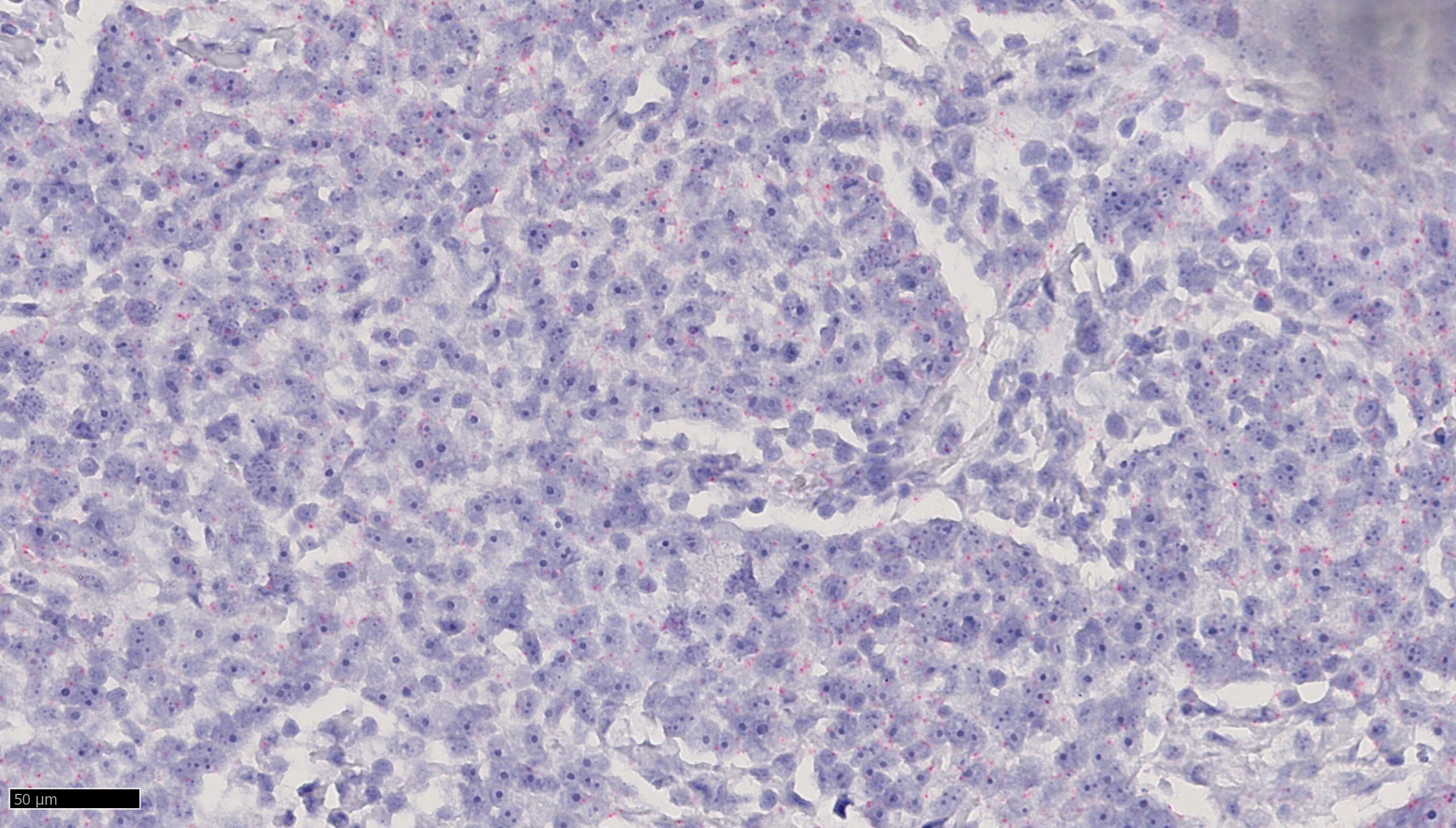

Supplement: S1 File — (ZIP) [file pgen.1011726.s002.zip › S2 figures - Kopi/Mamma pt2.jpg]

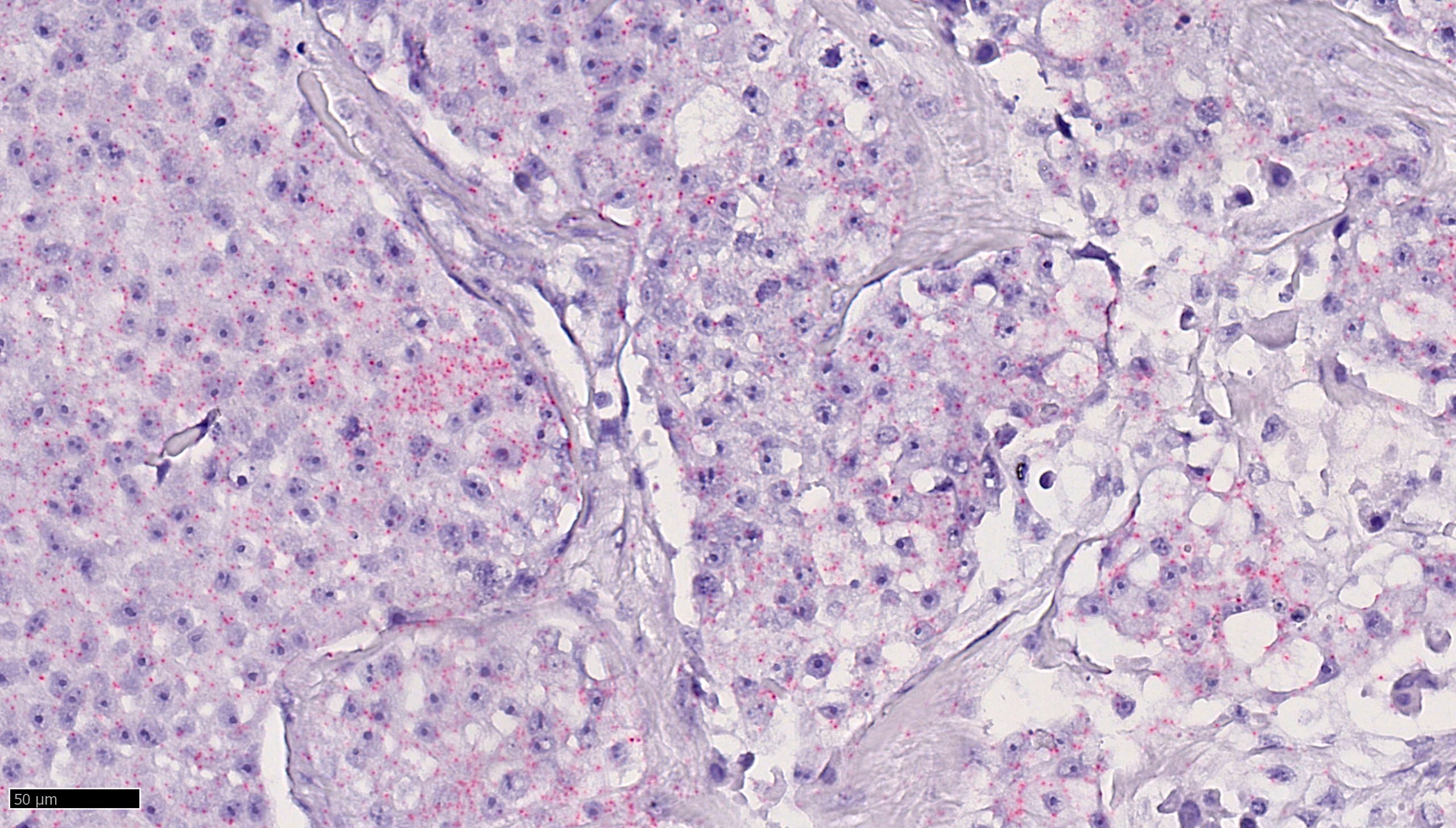

Supplement: S1 File — (ZIP) [file pgen.1011726.s002.zip › S2 figures - Kopi/Mamma pt3.jpg]

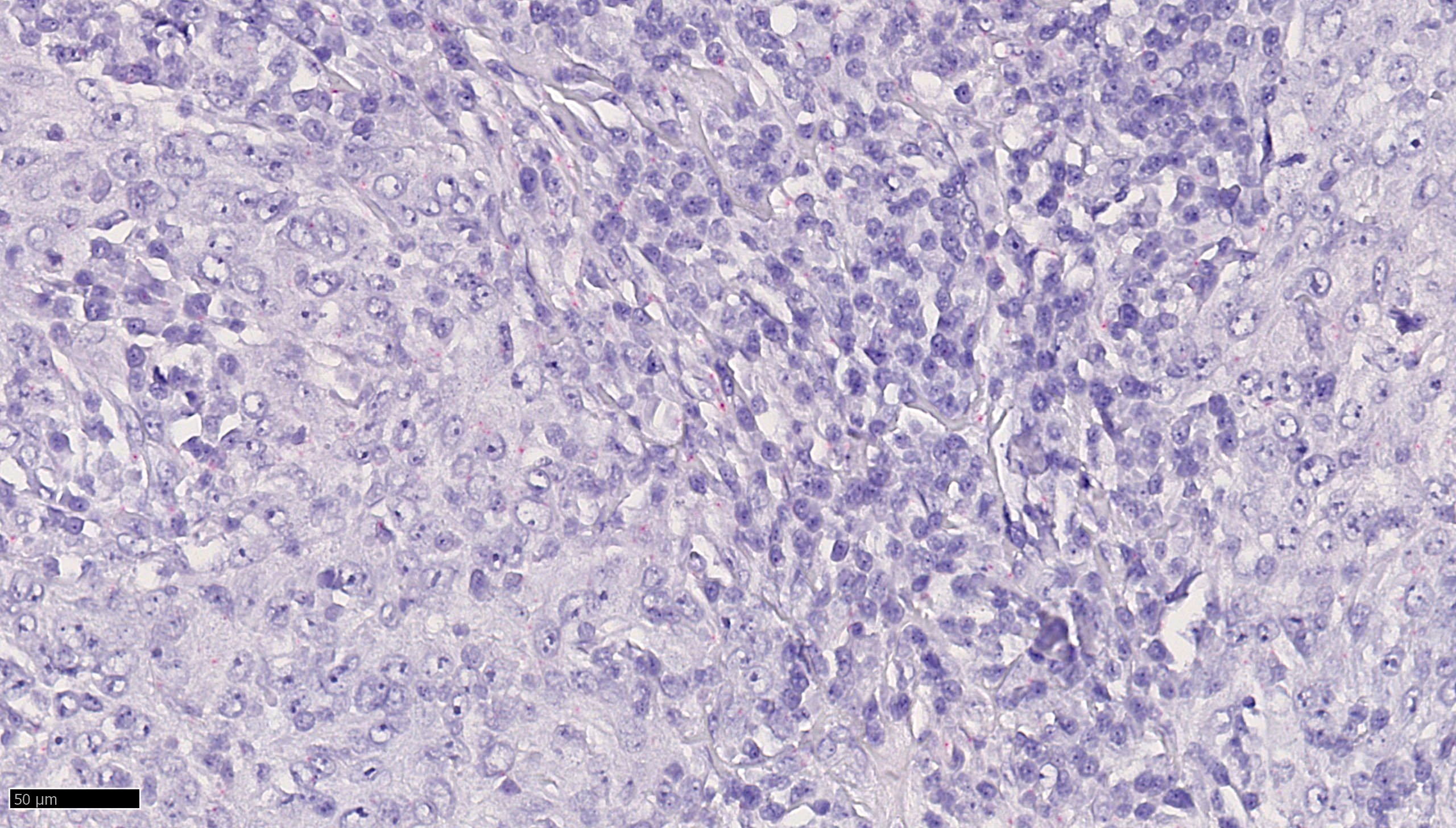

Supplement: S1 File — (ZIP) [file pgen.1011726.s002.zip › S2 figures - Kopi/Mamma pt4.jpg]

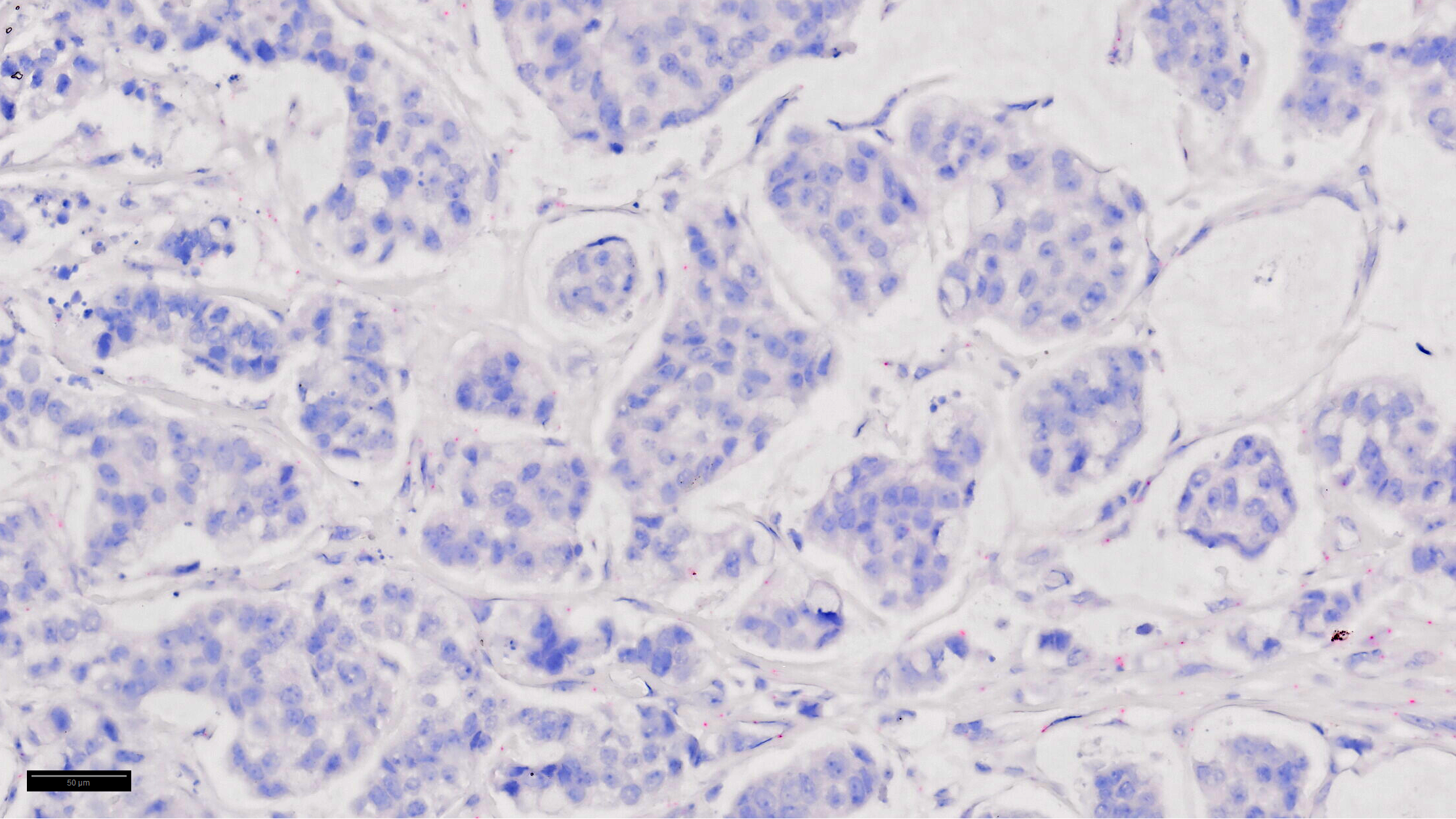

Supplement: S1 File — (ZIP) [file pgen.1011726.s002.zip › S2 figures - Kopi/Mamma pt5.tif]

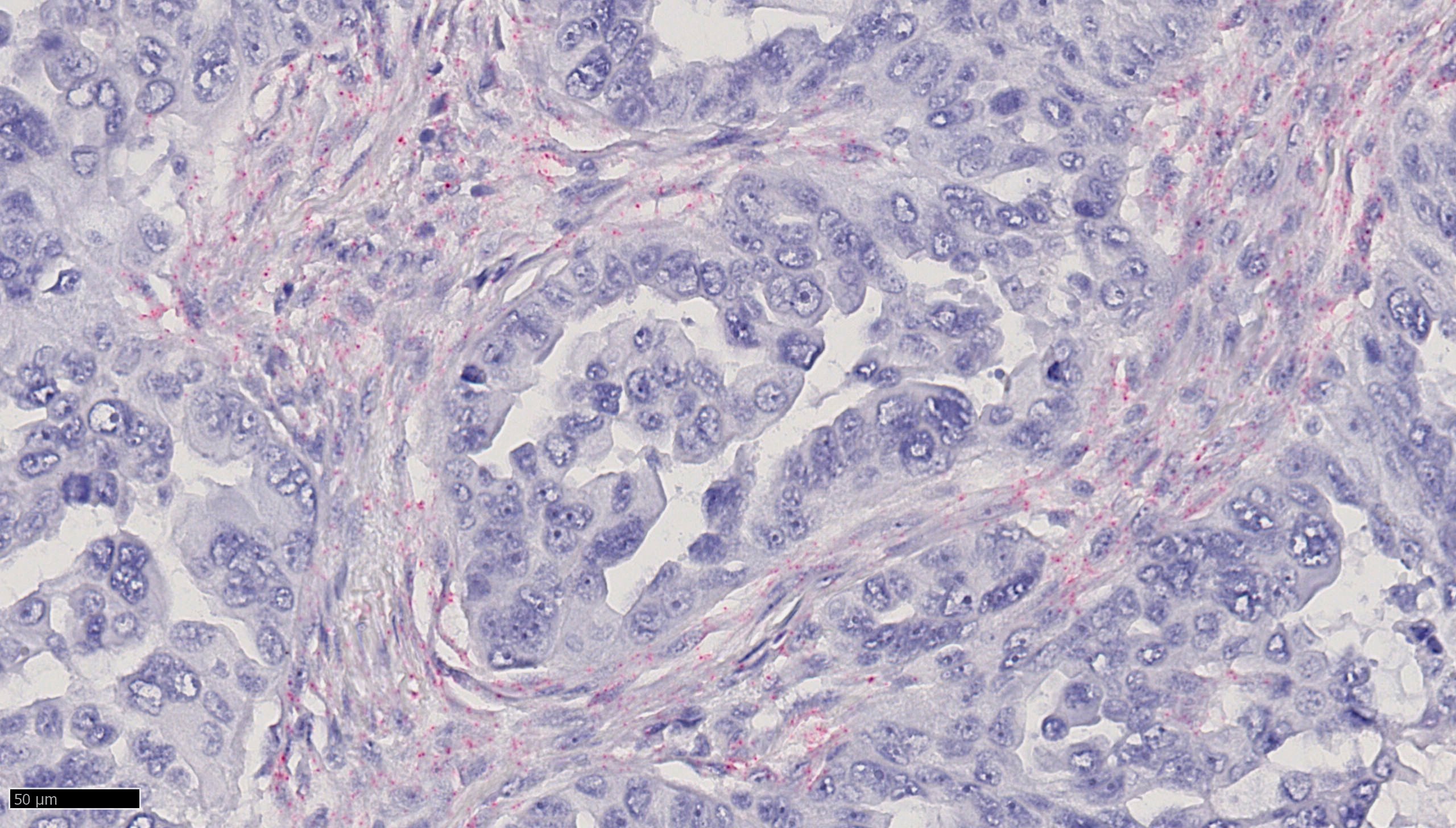

Supplement: S1 File — (ZIP) [file pgen.1011726.s002.zip › S2 figures - Kopi/Ovary pt1.jpg]

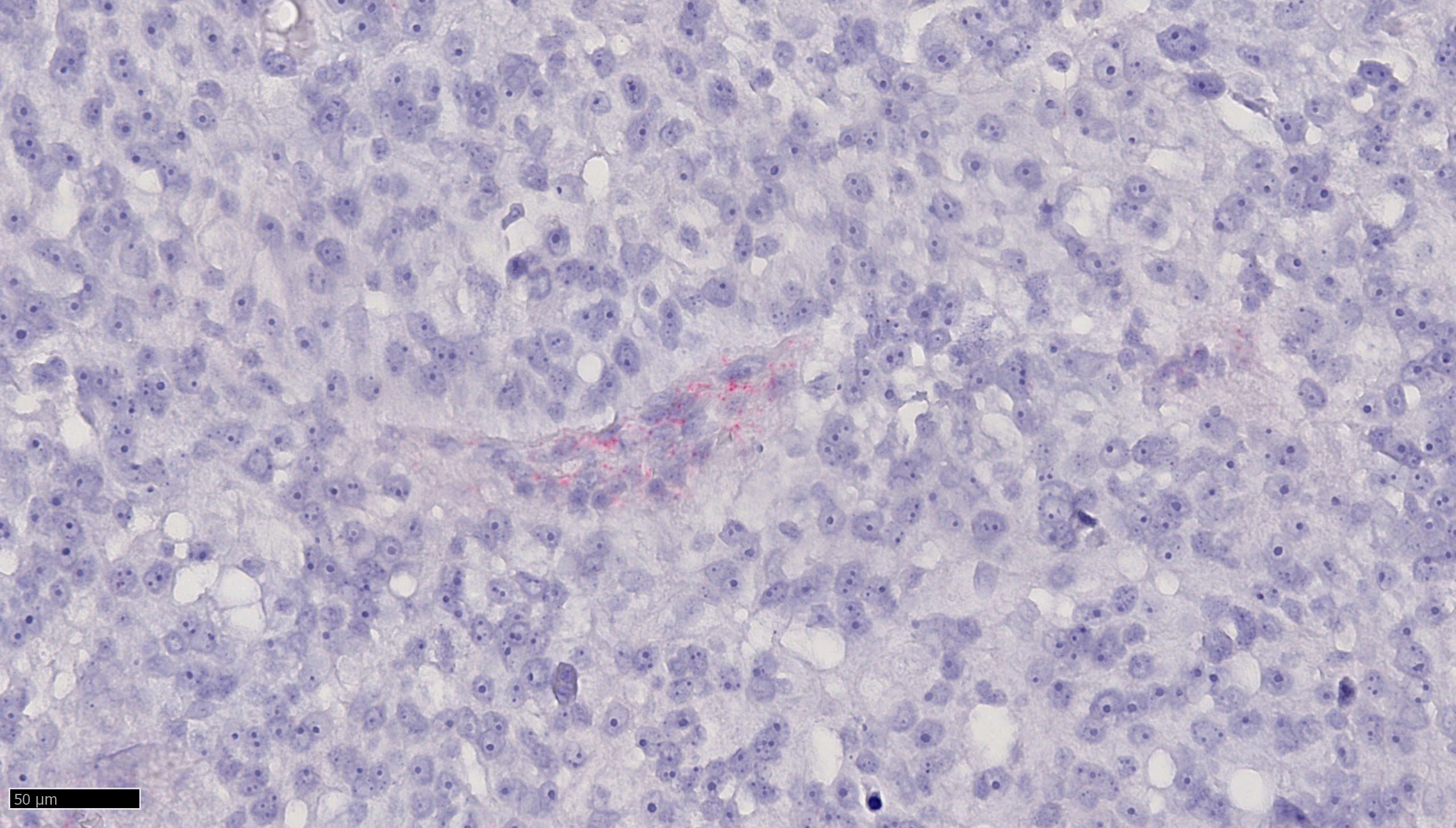

Supplement: S1 File — (ZIP) [file pgen.1011726.s002.zip › S2 figures - Kopi/Ovary pt2.jpg]

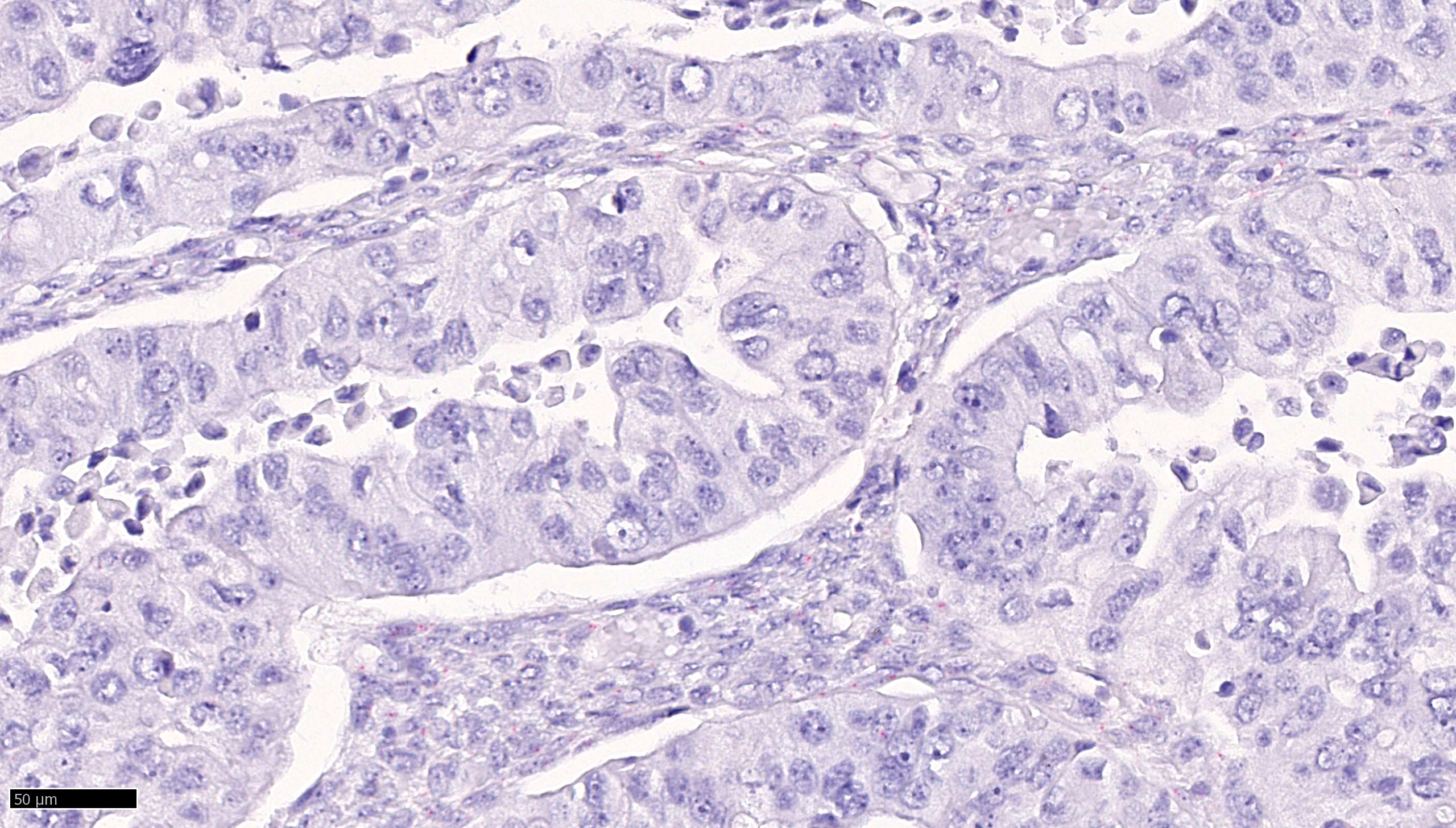

Supplement: S1 File — (ZIP) [file pgen.1011726.s002.zip › S2 figures - Kopi/Ovary pt3.jpg]

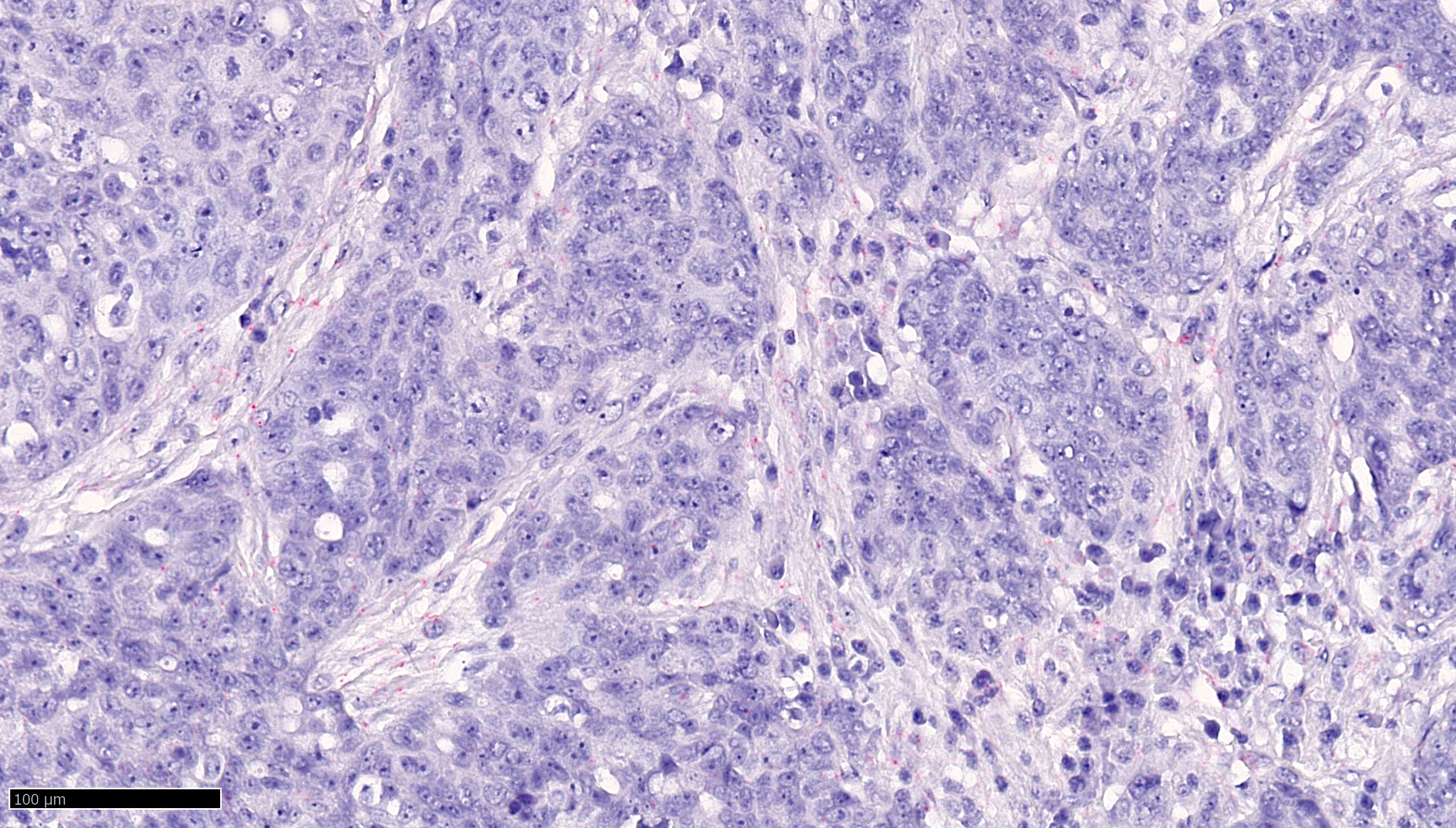

Supplement: S1 File — (ZIP) [file pgen.1011726.s002.zip › S2 figures - Kopi/Ovary pt4.jpg]

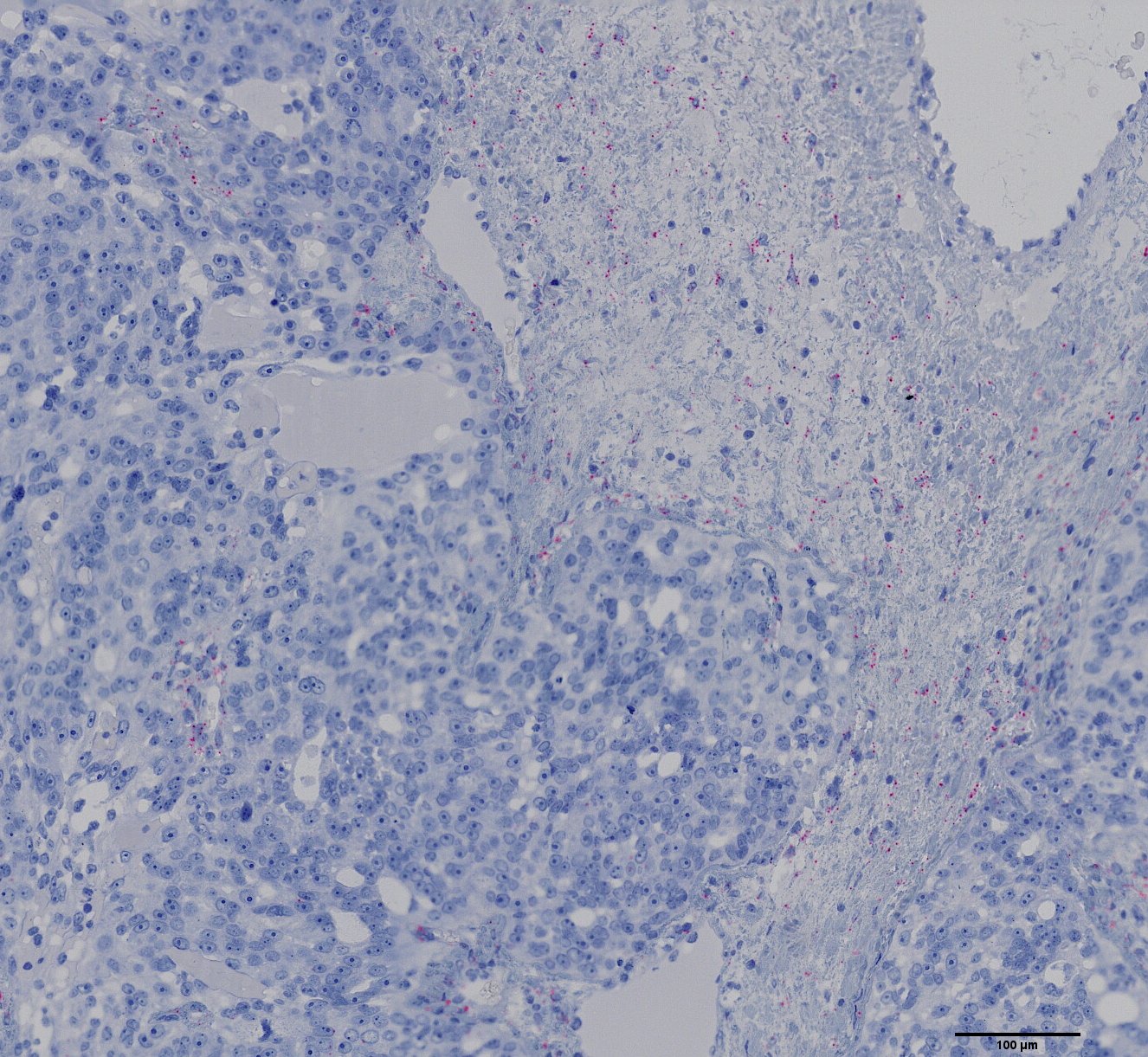

Supplement: S1 File — (ZIP) [file pgen.1011726.s002.zip › S2 figures - Kopi/Ovary pt5.jpg]

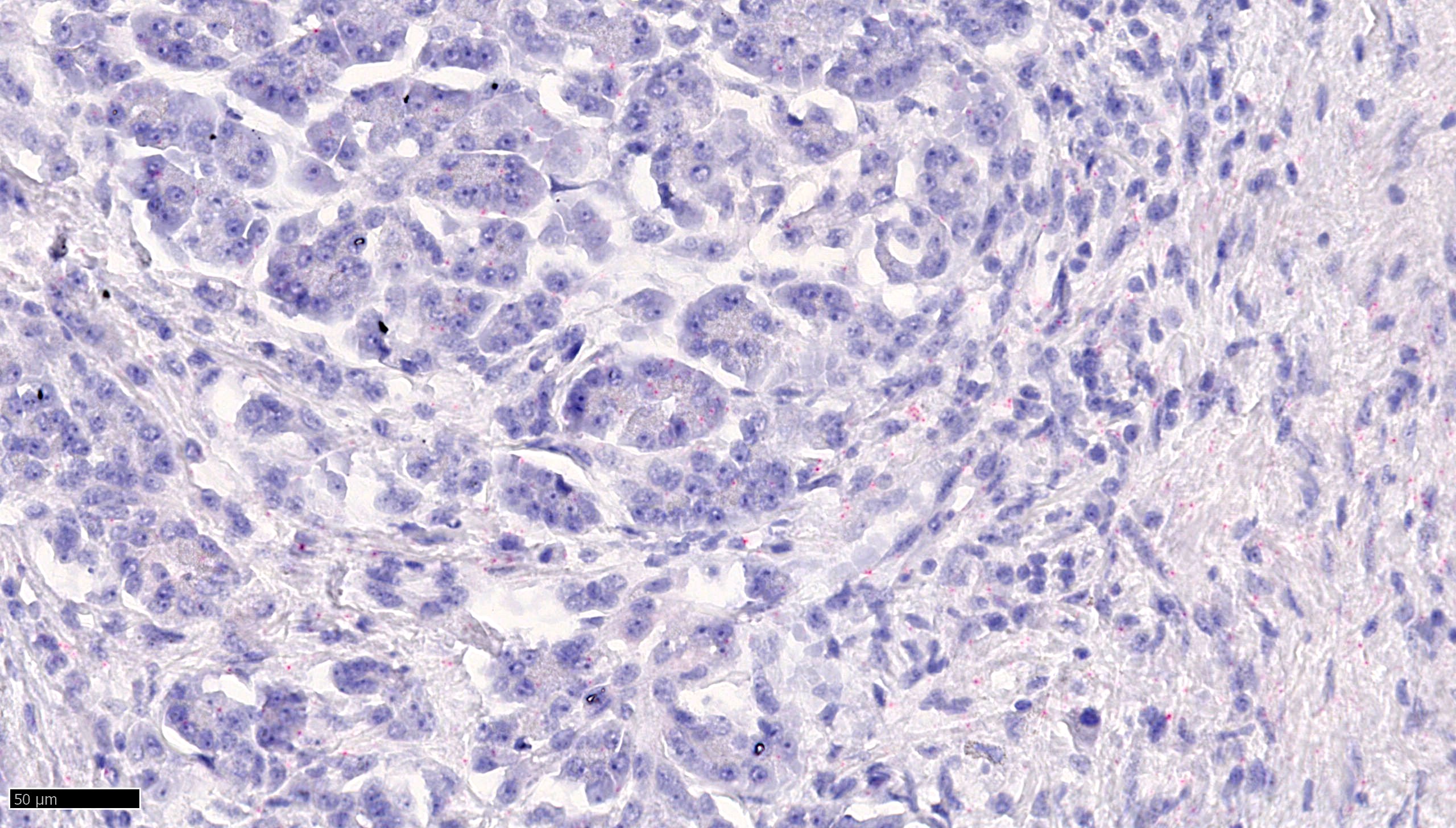

Supplement: S1 File — (ZIP) [file pgen.1011726.s002.zip › S2 figures - Kopi/Pancreas pt1.jpg]

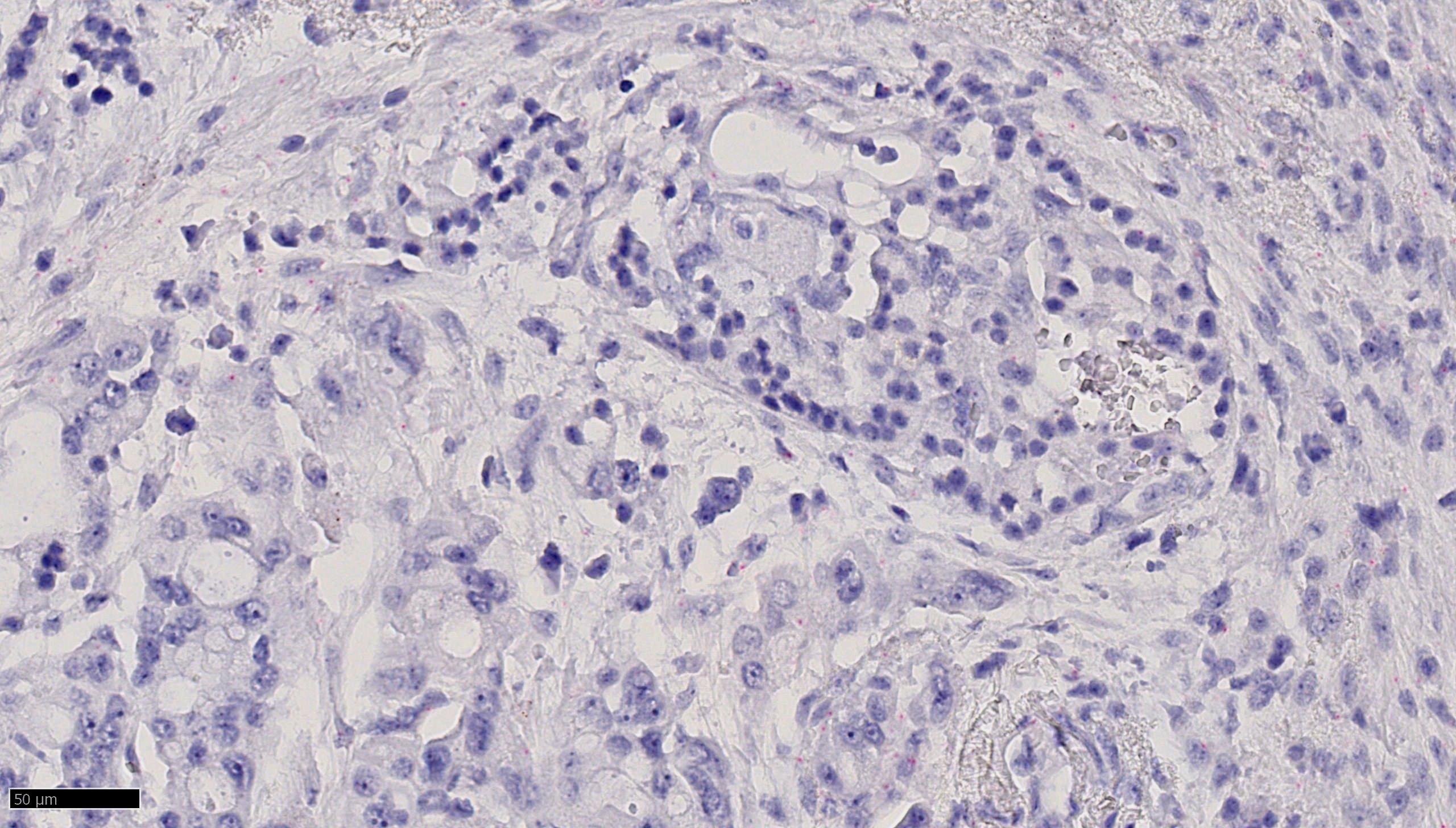

Supplement: S1 File — (ZIP) [file pgen.1011726.s002.zip › S2 figures - Kopi/Pancreas pt2.jpg]

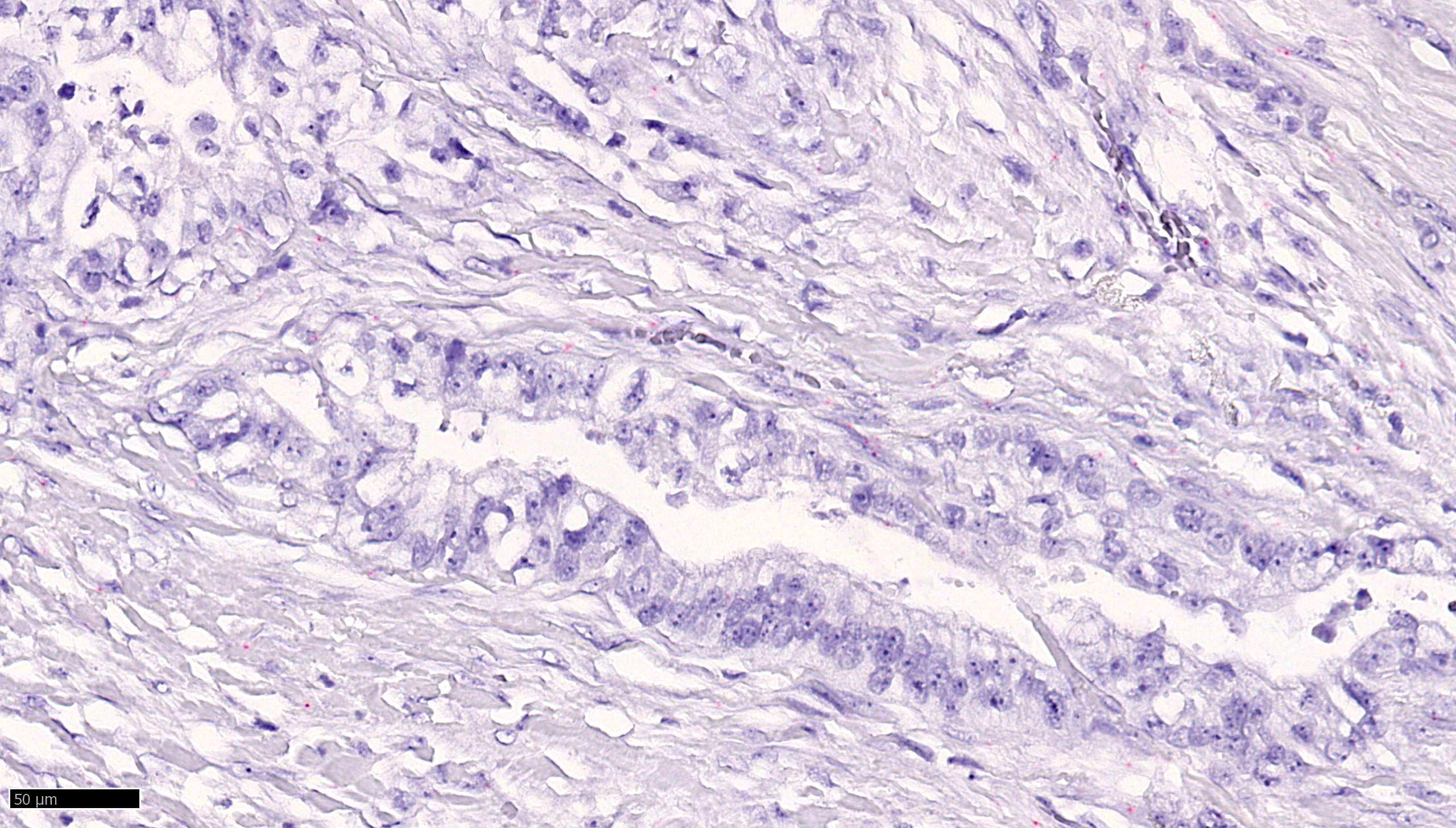

Supplement: S1 File — (ZIP) [file pgen.1011726.s002.zip › S2 figures - Kopi/Pancreas pt3.jpg]

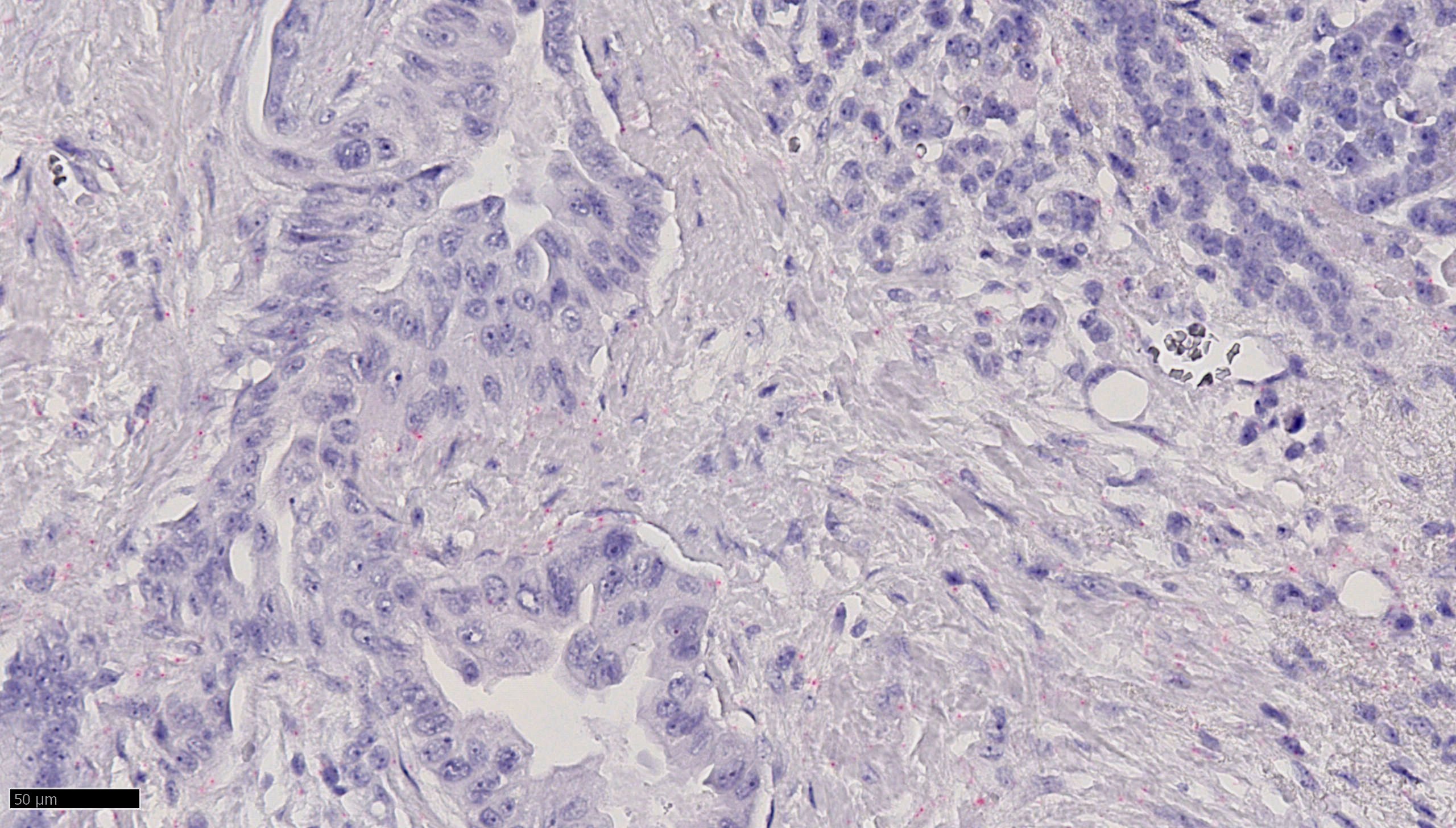

Supplement: S1 File — (ZIP) [file pgen.1011726.s002.zip › S2 figures - Kopi/Pancreas pt4.jpg]

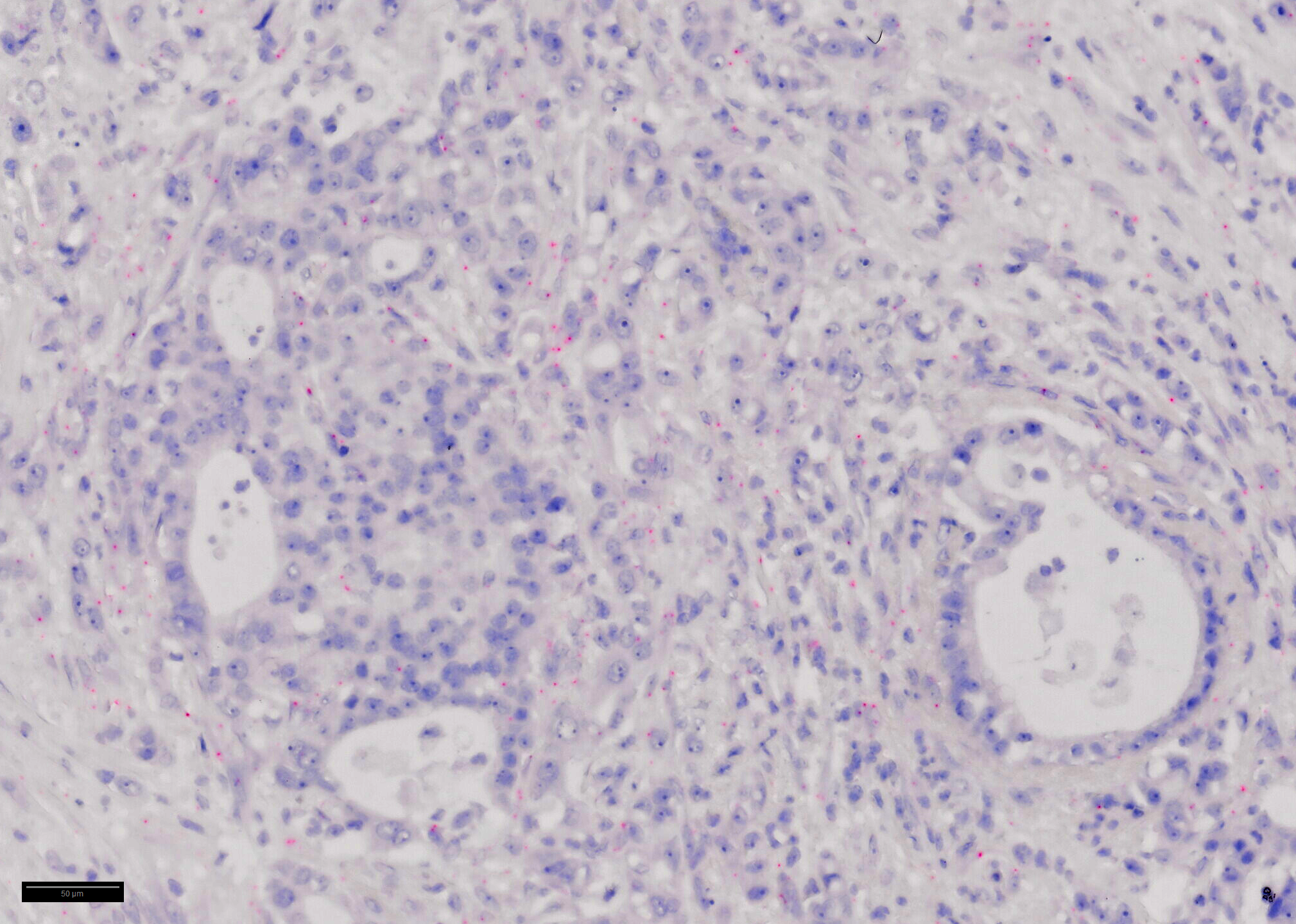

Supplement: S1 File — (ZIP) [file pgen.1011726.s002.zip › S2 figures - Kopi/Pancreas pt5.tif]

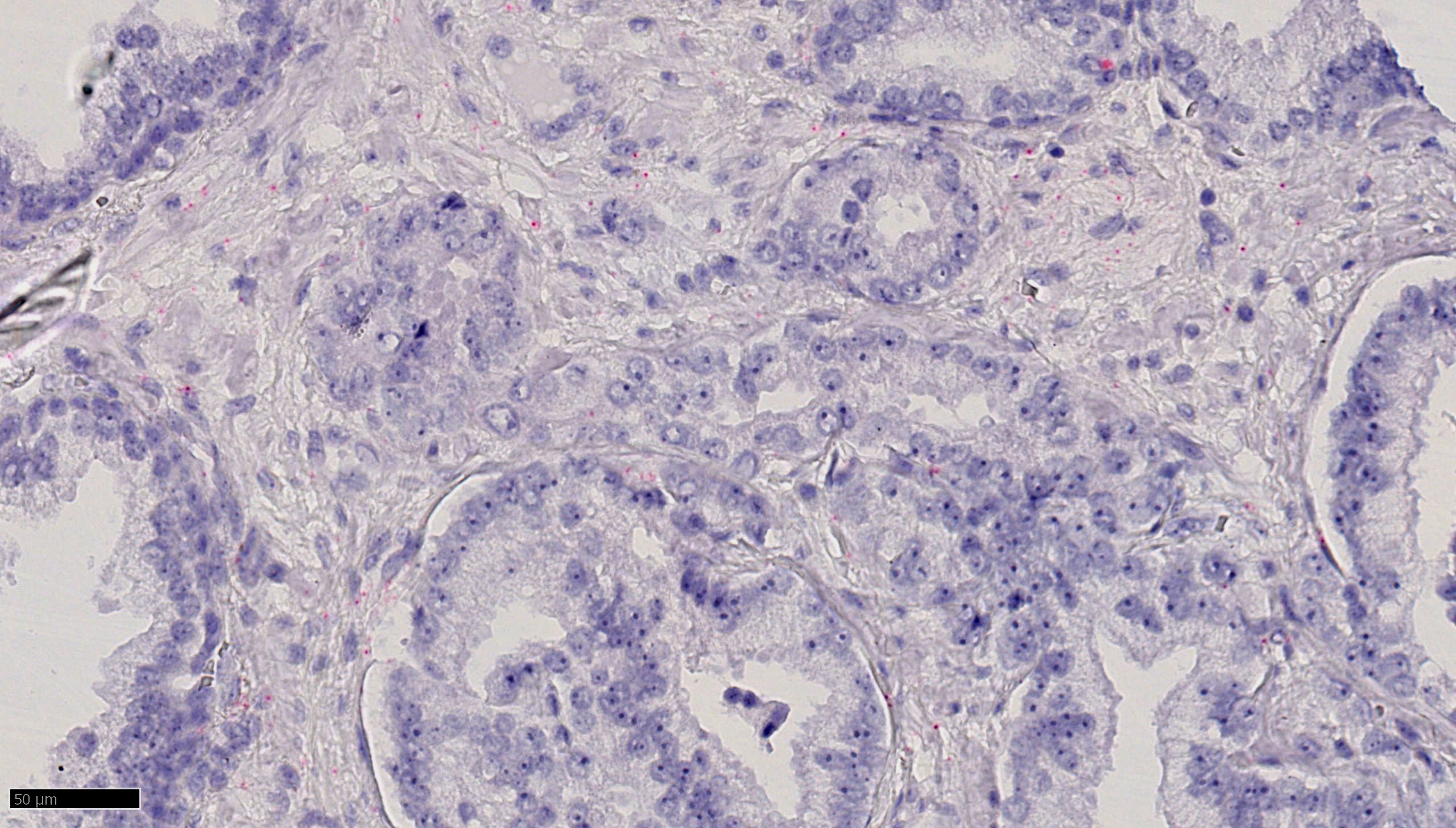

Supplement: S1 File — (ZIP) [file pgen.1011726.s002.zip › S2 figures - Kopi/Prostata pt1.jpg]

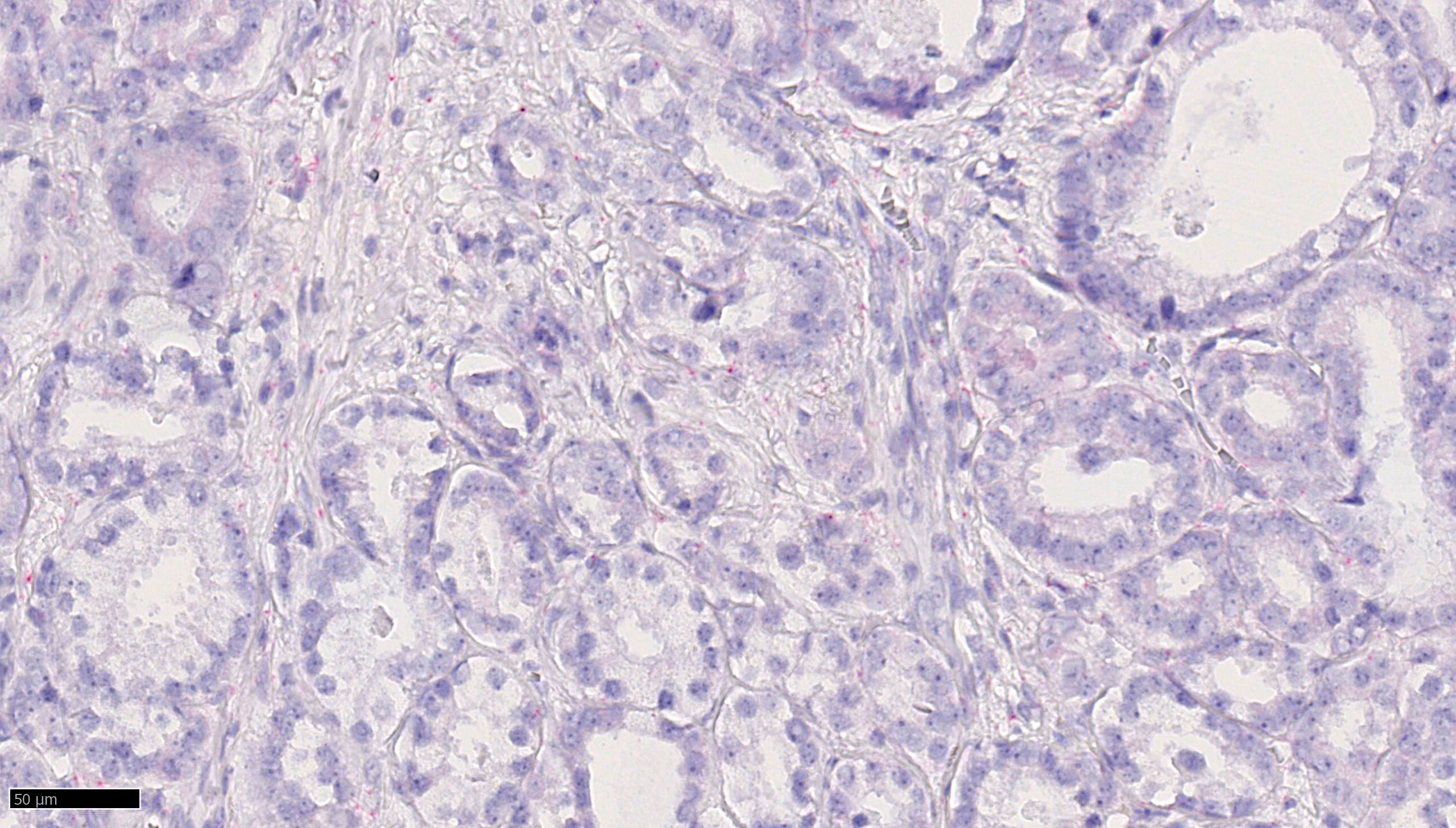

Supplement: S1 File — (ZIP) [file pgen.1011726.s002.zip › S2 figures - Kopi/Prostata pt2.jpg]

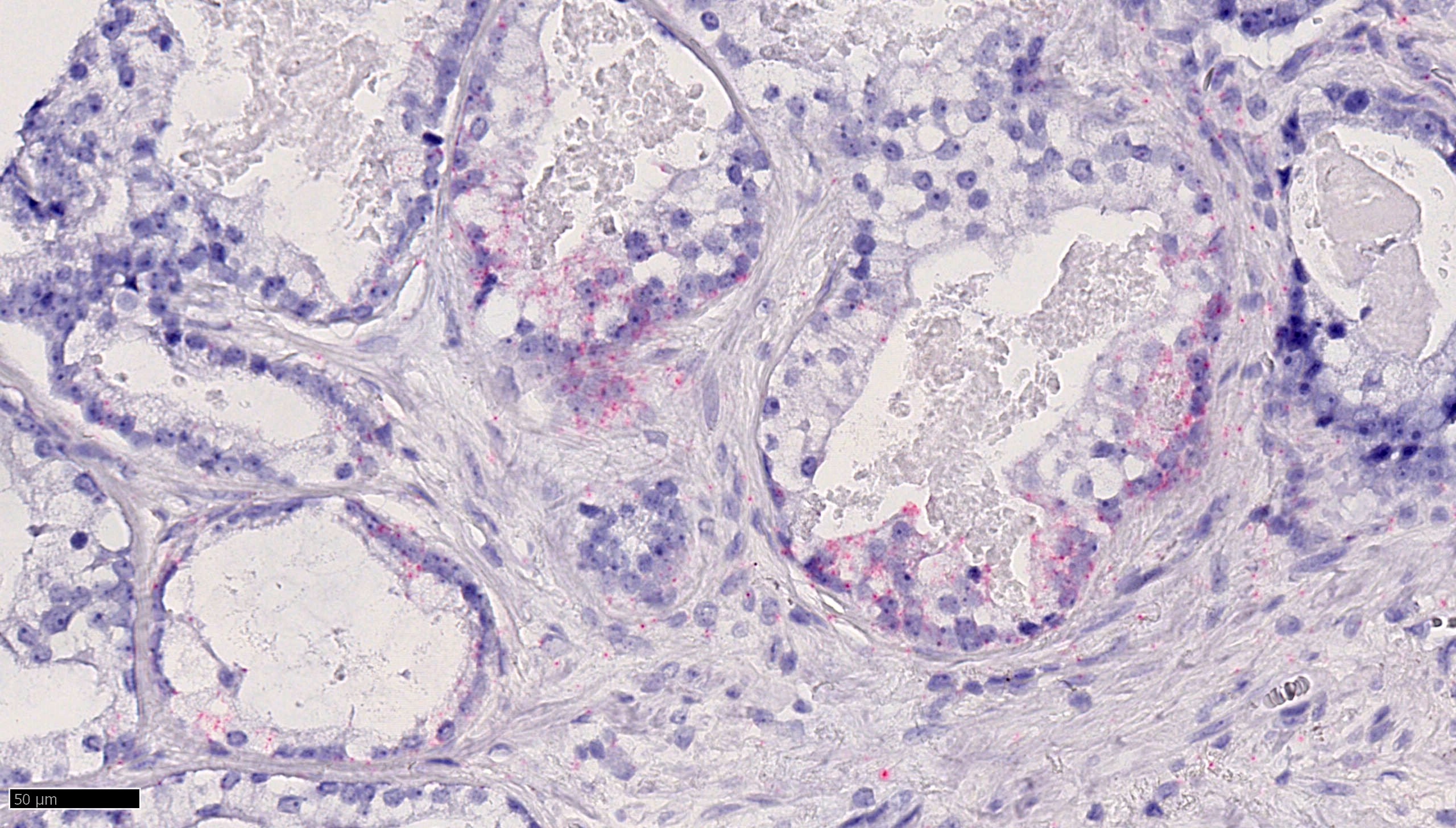

Supplement: S1 File — (ZIP) [file pgen.1011726.s002.zip › S2 figures - Kopi/Prostata pt3.jpg]

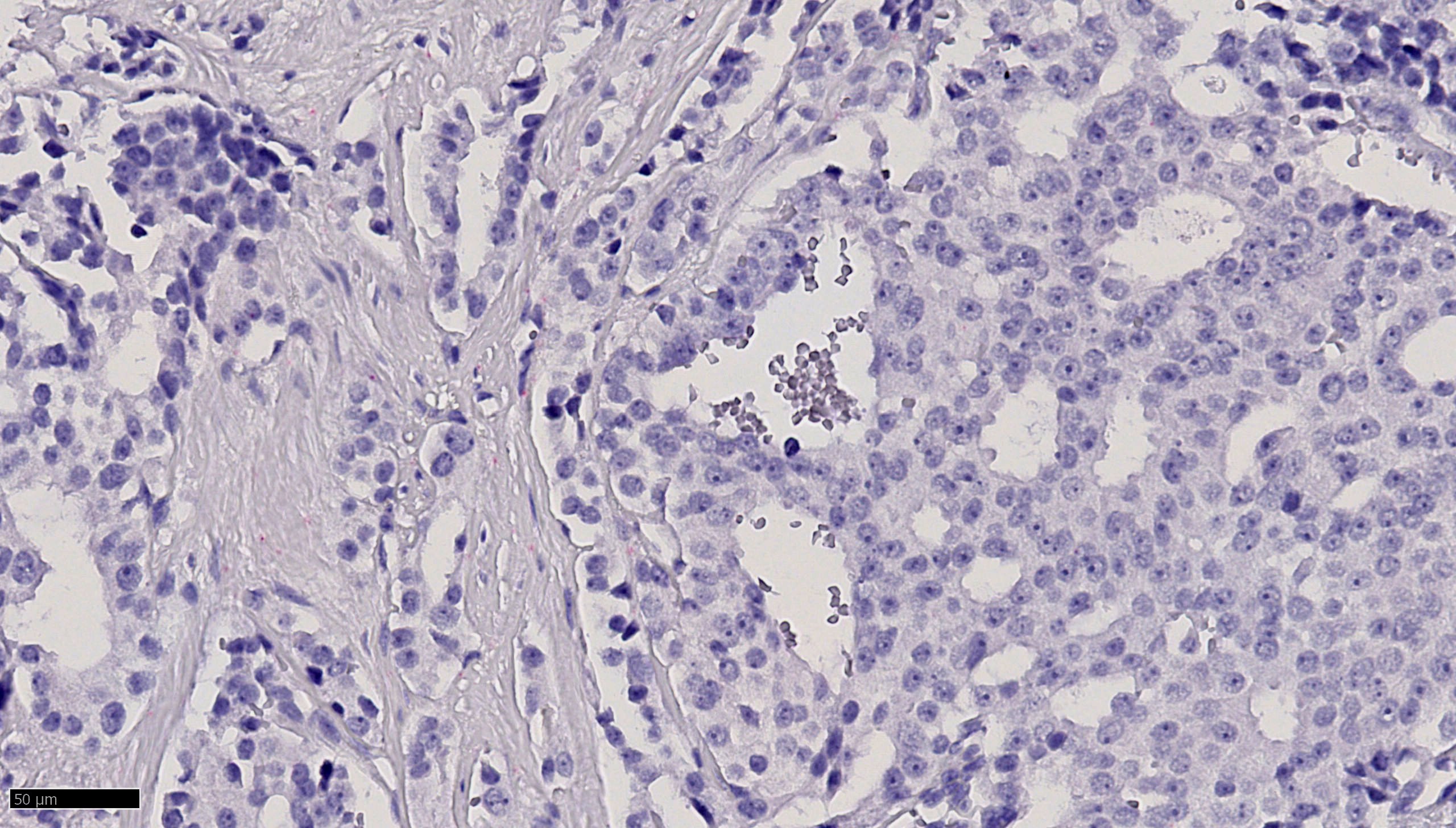

Supplement: S1 File — (ZIP) [file pgen.1011726.s002.zip › S2 figures - Kopi/Prostata pt4.jpg]

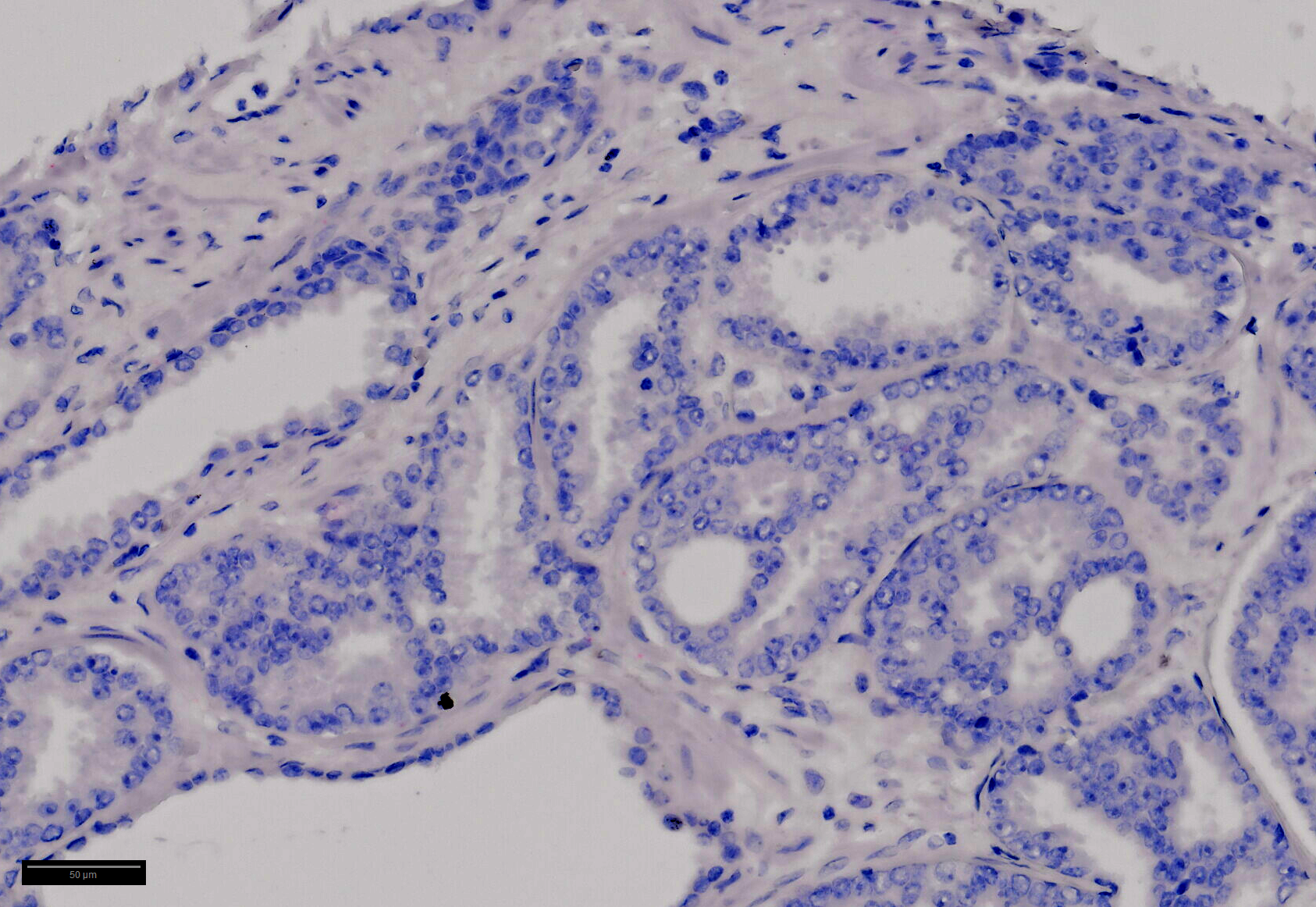

Supplement: S1 File — (ZIP) [file pgen.1011726.s002.zip › S2 figures - Kopi/Prostate pt5.tif]
