# Supplementary material for: ciRS-7 expression is epigenetically regulated in cancer cells across human adenocarcinomas
Source: PLoS Genet. 2025 Jun 2;21(6):e1011726. doi: 10.1371/journal.pgen.1011726 (PMC12162099; doi:10.1371/journal.pgen.1011726)
Supplement: S1 Table — (PDF) [file pgen.1011726.s004.pdf]

## Table S1: qPCR-primer list

| | |
|---|---|
| **T1 F** | GACATGGAATTCCTGGATCA |
| **T2 F** | CCGTCCCTTGCTGATTGTAT |
| **T1/T2 R** | GTCACTAGGTGGCAAACGTG |
| **T3 F** | GAGGCGGTTAAGGAGAGGAG |
| **T3 R** | TCAGGAGTCAAGGTCAGGCTA |
| **ciRS-7 F** | ACGTCTCCAGTGTGCTGA |
| **ciRS-7 R** | CTTGACACAGGTGCCATC |
| **GUSB F** | GCAGTACCATCTGGGTCTGGA |
| **GUSB R** | ACTCTCGTCGGTGACTGTTC |
| **UBC F** | CAGCCGGGATTTGGGTCG |
| **UBC R** | CACGAAGATCTGCATTGTCAAGT |
| **PUM1 F** | CATGCCAGGTTATCCGGTGT |
| **PUM1 R** | GCGCCTGCATTCACTACAAG |
