# Supplementary material for: ciRS-7 expression is epigenetically regulated in cancer cells across human adenocarcinomas
Source: PLoS Genet. 2025 Jun 2;21(6):e1011726. doi: 10.1371/journal.pgen.1011726 (PMC12162099; doi:10.1371/journal.pgen.1011726)
Supplement: S2 Table — (PDF) [file pgen.1011726.s005.pdf]

Table S2: nCounter gene panel

Gene	Probe target sequence (5’-3)
**ciRS-7**	AACGTCTCCAGTGTGCTGATCTTCTGACATTCAGGTCTTCCAGTGTCTGCAAT ATCCAGGGTTTCCGATGGCACCTGTGTCAAGGTCTTCCAACAACTCC
**T1**	GATCAAATGGAGGAGATGGAAGATTGAGAGCAGAGTGCAAGAATGGGAAG AGGGCTTCGTGCCCTGTGCACGTTTGCCACCTAGTGACCAAACTGAATGG
**T2**	TAGTTACCAGCAAGGCCTCTAGACCCATACTAACTGGGTTTGAATCAAGGTC CCGTCCCTTGCTGATTGTATCACCTTGGGAGGAGATGGAAGATTGAGA
**T3**	TGCAAAGACGAGACGAGTCCTCCCCAGAGAGGGGAGGCGGTTAAGGAGAG GAGATTGCGTGGCTCGGAGCAGCCAGACCCGATGGTGTGCTTCTCACCTC
**ACTB**	TGCAGAAGGAGATCACTGCCCTGGCACCCAGCACAATGAAGATCAAGATCA TTGCTCCTCCTGAGCGCAAGTACTCCGTGTGGATCGGCGGCTCCATCCT
**GUSB**	GGCCGCTGCTGGTGAAGGACTTCAACCTGCTTCGCTGGCTTGGTGCCAACGC TTTCCGTACCAGCCACTACCCCTATGCAGAGGAAGTGATGCAGATGTG
**MRPL19**	GGAAGTATTCTTCGTGTTACTACAGCTGACCCATATGCCAGTGGAAAAATCA GCCAGTTTCTGGGGATTTGCATTCAGAGATCAGGAAGAGGACTTGGAG
**PUM1**	CTGGGGAACATCAGATCATTCAGTTTCCCAGCCAATCATGGTGCAGAGAAG ACCTGGTCAGAGTTTCCATGTGAACAGTGAGGTCAATTCTGTACTGTCC
**SF3A1**	CTTCTAAGCCAGTTGTGGGGATTATTTACCCTCCTCCAGAGGTCAGAAATAT TGTTGACAAGACTGCCAGCTTTGTGGCCAGAAACGGGCCTGAATTTGA
