# Supplementary material for: ciRS-7 expression is epigenetically regulated in cancer cells across human adenocarcinomas
Source: PLoS Genet. 2025 Jun 2;21(6):e1011726. doi: 10.1371/journal.pgen.1011726 (PMC12162099; doi:10.1371/journal.pgen.1011726)
Supplement: S3 Table — (PDF) [file pgen.1011726.s006.pdf]

Table S3: ChIP-PCR primers

**LINC3 F (1)**	GCGTTCTGTGAAACTGAGCTT
**LINC3 R (1)**	AAGCTTTCAGAGGAGGACCG
**LINC3 F (2)**	CCCGTATTCCGCTTTAACCAA
**LINC3 R (2)**	AAGACGCTCCGCTCAGGGA
**LINC3 F (3)**	AAGGGCAAGCTCAGACCT
**LINC3 R (3)**	GGAATCACTTCAGAGTTGATTAGC
**UBC F (1)**	GTGATGCTTTTCTCCCCACTT
**UBC R (1)**	CAGGGTAGGGTGTCAAATATGA
**UBC F (2)**	CCGTCATTACCTCAAATGTGAC
**UBC R (2)**	CCTGCGAGATGGACGGGT
**RARβ1 F (1)**	GCCTGGCACATAATAGTGAGA
**RARβ1 R (1)**	GACCCATTTGTCTGTCTGCCT
**RARβ1 F (2)**	GTTGTAGTCTATGTTAGAATGTGAG
**RARβ1 R (2)**	CAGGTCTCCTAATTCCTTGTCTT
