# Supplementary material for: ciRS-7 expression is epigenetically regulated in cancer cells across human adenocarcinomas
Source: PLoS Genet. 2025 Jun 2;21(6):e1011726. doi: 10.1371/journal.pgen.1011726 (PMC12162099; doi:10.1371/journal.pgen.1011726)
Supplement: Attachment — Submitted filename: Response to reviewers.docx [file pgen.1011726.s009.docx]

*Dear Editors.*

*We thank you and the reviewers for the helpful comments and suggestions on our manuscript entitled “ciRS-7 expression is epigenetically regulated in cancer cells across human adenocarcinomas”.*

*Please find hereafter the detailed response to all comments as well as attached the revised manuscript with and without track changes. We have addressed the concerns raised and edited the manuscript accordingly. Additionally, we have identified and corrected a few additional mistakes.*

*We believe that the incorporated changes improved the manuscript substantially and hope you will find the revised version satisfactory as well.*

**Reviewers' comments:**

**Reviewer's Responses to Questions**

**Reviewer #1: The study first analyzed ciRS-7 expression levels and spatial distribution in nine types of adenocarcinomas and 18 cell lines, revealing a positive correlation between ciRS-7 expression and the T3 transcript of LINC00632. Further investigations demonstrated that DNA methylation and H3K9 acetylation were critical epigenetic modifications regulating ciRS-7 (LINC00632) expression, while H3K27 methylation showed no significant impact. Through inhibitor experiments, the regulatory roles of H3K9 acetylation and DNA methylation on ciRS-7 expression were confirmed. Additionally, using laser-capture microdissection to distinguish cancer cells from the tumor microenvironment (TME), the study established that ciRS-7 expression was predominantly localized in the TME. However, I have some questions regarding the research content:**
**1. Fig. 1: How many samples were used for tissue FISH validation of ciRS-7 in each type of adenocarcinoma? If only one sample was analyzed for each cancer type, it might not be sufficient to conclude that ciRS-7 is not expressed in a particular cancer.**

We appreciate that the reviewer has clearly understood the message of our study.

We thank the reviewer for the insightful comment and agree that analyzing only a few samples per cancer type is not sufficient to conclude that ciRS-7 is not expressed in a particular type of cancer. Therefore, we have included more patient samples for all the cancer types (breast (n=5), pancreas (n=5), prostate (n=5), lung (n=5), colon (n=5), endometrium (n=5), ovary (n=5), bile duct (n=5), and stomach (n=4)). The main figure, the text and the method section have been updated accordingly. These analyses revealed that ciRS-7 is expressed within the cancer cells in a few cases. Related to this, a new figure has been included (Supplementary Figure S1) and the related text updated in the new version of the manuscript.

**2. Although LINC00632 is the host gene of ciRS-7 and positively correlated with ciRS-7 expression, Fig. 2F indicates that H3K27me3 does not show significant differences in LINC00632 (T3). Thus, the statement in Line 172–173, "H3K27me3 was present in all cell lines (Fig. 3F) despite the large differences in ciRS-7 expression," is not accurate and needs revision.**

We thank the reviewer for this comment, however, it is related to Fig. 3F and not Fig. 2F. Nevertheless, we agree that this comment should be revised. This has now been corrected, to reflect the results more precisely, and the sentence now reads “H3K27me3 was present in all the analyzed cell lines (Fig. 3F,) despite their differing ciRS-7 levels”.

**3. The FFPE colon cancer patient samples in Fig. 5 include only three samples, which raises concerns about potential sampling bias.**

As Laser Capture Microdissection, as well as the subsequent analyses, are very labor intensive, we had initially included three patients. However, we agree with the reviewer that the low number of patients could potentially create a sampling bias. Therefore, as suggested, we have now increased the number of patient samples to have a total of five samples analyzed for each fraction for both ciRS-7 expression and DNA methylation levels. These data were in line with the previous data and thus the interpretation of data has not changed. We have updated the figure as well as the related text accordingly.

**4. The key finding of this study was that the transcription of LINC00632 was regulated by epigenetic modifications. As the host gene of ciRS-7, LINC00632 may have influenced ciRS-7 expression. Therefore, ciRS-7 itself may not have been directly regulated by histone modifications.**

Studies have shown that LINC00632 is always responsible for the generation of ciRS-7 (PMID: 29236709), as ciRS-7 does not have another separate promoter region. Moreover, the backsplicing of ciRS-7 is a very efficient process (PMID: 32683316). Thus, we will argue that the statement regarding the epigenetic regulation of ciRS-7 still applies in this context.

**Spelling error:
Line 217: "adenocarcinoma patient samples (Fig. 5. C)" need corrected to " ...(Fig. 5C)".**

Thank you for your careful review and for pointing out this typo, which has now been corrected.

**Reviewer #2: In this study, the Authors investigated the expression and epigenetic regulation of the circular RNA ciRS-7 across 9 types of adenocarcinomas. They demonstrate that ciRS-7 is absent in cancer cells but expressed in the tumor microenvironment (TME). Moreover, epigenetic modifications, specifically DNA methylation and H3K9 acetylation, were found to regulate ciRS-7 expression. This is an interesting and thorough study that provides insights into the role of ciRS-7 in cancer biology and its potential as a therapeutic target. However, there are several issues in the manuscript:**

We would like to thank the reviewer for stating that we have provided an interesting and thorough study.

**1. While the presence of ciRS-7 in the TME is well-described, the manuscript lacks experimental evidence on its specific functional roles. Additional experiments or cited literature linking ciRS-7 in the TME to tumor progression, immune modulation, or therapeutic resistance would significantly strengthen the conclusions of the study.**

We agree with the reviewer that this manuscript does not comment on the specific functional roles of ciRS-7. This manuscript mainly focuses on the epigenetic regulation of ciRS-7. Thus, no functional studies were performed as this was deemed to be outside the scope of this current manuscript. However, we agree that this manuscript would benefit from a discussion of the potential functional role of ciRS-7, and therefore we have expanded the discussion section to cover this and cited additional relevant literature.

**2. The Methods section should include more details, for instance regarding normalization strategies for RT-qPCR and nCounter data, and sample size limitations for certain experiments.**

Thank you for this comment. We agree that the method section should include as many details as possible regarding normalization and sample size limitations to increase reproducibility of the experiments. As suggested by the reviewer, more details have been added to the method section. In addition, we have included additional patient samples to address sample size limitations for the FISH experiment (Fig. 1 and Supplementary Figure S1 in the new version of the manuscript) and the experiments performed on the microdissected tissues (Fig. 5 in the new version of the manuscript).

**3. The absence of ciRS-7 in cancer cells is demonstrated. Did the Authors address variability across cancer subtypes within adenocarcinomas? Is there any data regarding heterogeneity in ciRS-7 regulation across tumor stages or grades?**

We have performed experiments on additional patient samples and as suggested by the reviewer, we have addressed the differences in ciRS-7 expression across the adenocarcinoma subtypes. Clinical information regarding the patient samples was, unfortunately, not available.

**4. The Discussion could include more in-depth commentary. For example, the potential oncogenic role of ciRS-7 in TME is briefly mentioned, but the Authors do not explore therapeutic targeting strategies. Discussion on targeting epigenetic modifiers or disrupting ciRS-7 interactions would provide translational relevance.**

We thank the reviewer for this insightful comment. Although the main scope of this paper is exploring the epigenetic regulation of ciRS-7 expression, we agree with the reviewer that the paper would benefit from a discussion of the potential therapeutic targeting strategies of ciRS-7. We have added a section in the discussion where this is explored and cited new relevant articles related to this subject.

**5. It would be interesting to also discuss the potential role of ciRS-7 as a non-invasive biomarker. The Author could refer to recent studies that suggest its diagnostic/prognostic role in cancer, such as: Papatsirou et al., EJHaem. 2024;5(4):677-689.**

We thank the reviewer for this great suggestion and have included this subject in the section described above now also citing the suggested article.

**Reviewer #3: In this manuscript, Paasch and coauthors characterize the expression of ciRS-7 in different tumor contexts. While absent in most adenocarcinoma cells, it was found in the tumor microenvironment. Additionally, the authors investigate the epigenetic mechanisms that regulate ciRS-7 expression.

The specific localization of ciRS-7 within the microenvironment rather than in cancer cells was unexpected, providing valuable insights into the relevant milieu for circRNA function. The study is well-conducted and clearly presented, although a more in-depth exploration of ciRS-7's role specifically in the stroma would be beneficial.**

We would like to thank the reviewer for stating that our study is well-conducted and clearly presented, as well as providing valuable insights into the milieu for circRNA function.

**Major points**
**1. The correlation between T3 and ciRS-7 expression is based on only a few cell lines (Figure 2). To further substantiate the link between these two RNAs, T3 transcript expression should also be analyzed in Figure 4 following** **5-aza and SAHA treatment.**

We thank the reviewer for this comment, and we kindly highlight that the correlation between T3 and ciRS-7 in figure 2 is based on 18 different cell lines and significant despite T3 and ciRS-7 expression is very low or absent in most cell lines. However, as suggested by the reviewer, we have included T3 data for the experiments using 5-aza and SAHA treatment (See revised version of Figure 4). In line with the ciRS-7 data, we also observe increased levels of the T3 transcript upon the treatments.

**Similarly, are T1-T3 also differentially expressed between adenocarcinoma cells and the stroma (Figure 1)?**

Although the reviewer has posed an interesting question, these transcripts are very likely to be unquantifiable using FISH, as they would be under the limit of detection for the assay, in line with our qPCR results, where the T1 and T2 transcripts were under the limit of detection as well. This is most likely due to the efficient backsplicing process of LINC00632 leading to the formation of ciRS-7(PMID: 32683316).

**Minor points**

**1. Line 165: Should read “…as well as in two cell lines with no ciRS-7 expression and high...”.**

Thank you for your careful review and for pointing out this typo. We have corrected the sentence as suggested.

**2. Line 170: Typo correction: "A459" should be "A549**."

Thank you for your careful review and for pointing out this typo, which has now been corrected.

**3. Figure 3E: Only two replicates are shown for the H1650 cell line. For robustness, please include a third replicate.**

We thank the reviewer for this comment and agree that it seems that only two replicates are shown, but a third replicate was indeed included in the original figure. However, as two of the three datapoints are very close, they are almost placed on top of each other and therefore look like a single point.

**4. Figure 4: To better interpret the extent of reactivation upon treatment with different epigenetic modulators, expression levels should be normalized to baseline (conc=0).**

As suggested by the reviewer, we have compared the expression levels of ciRS-7 to the negative control sample using the $2^{-\Delta\Delta Ct}$ method (see figure below). However, when transforming the data points and comparing these to the negative control (which is set to 1), variability in this group is diminished, which influences the subsequent statistical tests. Therefore, we prefer to show our data as in the original version of the manuscript, where the variability of the negative control sample is shown.

**5. Lines 262-263: Please review and revise the syntax for clarity.**

Thank you for your careful review and for pointing out this typo. The lines have now been revised and the syntax has been updated to enhance the clarity of the text.
